# Post-Layout Symmetry and Trade-Offs in a CMOS Hysteresis Comparator

**DOI:** 10.3390/mi17101135

**Published:** 2026-09-29

**Authors:** Petru-Alexandru Ștefănescu, Lidia Dobrescu

**Affiliations:** Devices, Circuits and Electronic Architectures Department, National University of Science and Technology POLITEHNICA Bucharest, 060042 Bucharest, Romania; petru.stefanescu99@stud.etti.upb.ro

**Keywords:** CMOS comparator, hysteresis, symmetry, analog layout, parasitic extraction, charge pump

## Abstract

Analog layouts often use matched devices to reduce sensitivity to process gradients. However, the resulting interconnect can introduce parasitic coupling. We compare four DRC-clean, LVS-equivalent layouts of the same hysteresis comparator, implemented in a 130 nm bulk CMOS process with 5 V, thick-oxide devices. The schematic, bias, load, and R+C+CC extraction flow are held fixed, while placement and routing vary together. At 5.0 V and 25 °C, the strictly common-centroid layout has a simulated 315.758 mV high-state output ripple despite nearly balanced input capacitances. Among the protected layouts, C3 yields the narrowest finite-slew switching window, whereas C4 reduces nominal ripple to 0.275 mV. In 200 mismatch samples at the nominal 5.0 V supply, C3 and C4 have similar threshold dispersion, while C4 retains lower output ripple. The observations support evaluating placement and routing together with device matching. The preferred layout depends on the chosen performance metric.

## 1. Introduction

Geometrical symmetry is widely used in analog layout, where common-centroid placement, mirroring with respect to a central axis or point, and device interdigitation are commonly employed to improve matching and to reduce the influence of spatial process and mechanical variations. However, the same placement strategies often force the corresponding interconnections to remain close to each other, to cross between different metal layers, and in some cases to follow longer and more interleaved routing paths, not only among the matched branches themselves but also with respect to neighboring signal lines. Therefore, while the device geometry is arranged to preserve matching, the associated routing may introduce an additional electrical penalty through increased parasitic resistance, capacitance to the substrate, and coupling capacitance between the routed nodes and other signal-related nets.

High-voltage generators and non-volatile-memory interfaces use charge pumps to provide the voltages required for EEPROM programming and erasing, while a regulation loop limits ripple and electrical stress by comparing the monitored pump voltage with a reference and controlling the pumping activity [1,2]. Thus, a hysteresis comparator is well suited to this interface because its distinct rising and falling thresholds suppress repeated transitions near the regulation point, although its switching window, center, propagation behavior, and susceptibility to coupled disturbances depend on both the regenerative circuit and the post-layout parasitics [3,4]. Recent comparator studies have accordingly emphasized operating-range and robustness validation: Yao and Jiang evaluated five process corners and three supplies [5], while Della Sala et al. used post-layout PVT simulations and a 100-run mismatch analysis [6]. These studies provide relevant validation references, but they compare circuit architecture rather than alternative layouts of one fixed schematic.

From the point of view of the schematic, corresponding input and bias devices must be matched because differences in threshold voltage, mobility-related parameters, or effective geometry produce input-referred offset and unequal branch currents [7], while the Pelgrom relation explains the area dependence of local random mismatch [8]. Common-centroid patterns, proximity, matched orientation, interdigitation, and dummy completion are standard methods for suppressing first-order spatial gradients [9,10,11], and hierarchical techniques can identify the symmetry constraints embedded in repeated circuit structures [12].

The limitations of geometric matching are already recognized. Sharma et al. described the routing and parasitic costs of common-centroid layouts [11] and subsequently optimized common-centroid placement together with parasitic-aware routing and layout-dependent effects [13]. Maji et al. investigated nonlinear spatial variation in non-common-centroid placement [14] and multiobjective common-centroid placement optimization [15]. Layout-aware sizing and synthesis have likewise incorporated parasitic estimates into the design loop [16,17]. These works establish that strict common-centroid geometry is not universally optimal. Our narrower contribution is an electrical comparison of four completed, DRC-clean, LVS-equivalent implementations of one comparator under a fixed schematic, bias, load, and extraction flow; we evaluate switching behavior and ripple alongside the extracted interconnect.

This work therefore does not claim to introduce a new placement algorithm or to discover the general limitations of common-centroid matching. It tests how different completed physical implementations of the same 5 V comparator trade finite-slew switching-window width against high-state output ripple. Placement and its resulting routing change together, so this comparison does not isolate their individual effects.

The controlled comparison retains the same circuit, transistor dimensions, bias, load, and extraction procedure across C1–C4. Extracted input and internal-node parasitics are examined alongside switching thresholds, finite-slew hysteresis, and output ripple. C2 illustrates an unfavorable response despite strict geometric matching; among layouts with the same dummy and guard-ring environment, C3 has the narrower nominal switching window and C4 has the smaller nominal output ripple. These observations compare complete layout solutions rather than attributing a measured difference to one isolated geometric feature.

## 2. Comparator Architecture and Operating Principle

### 2.1. Overall Comparator Architecture

The investigated circuit is a continuously biased CMOS hysteresis comparator containing a differential input stage, a second gain stage, a regenerative network, and a two-inverter output buffer. It represents the sensing interface of an EEPROM charge-pump regulation loop, while the pump and the circuit that generate the monitored voltage remain outside the present scope.

The non-inverting and inverting inputs are denoted P and M, with voltages *V*_P_ and *V*_M_; P receives a filtered reference of approximately 1.04 V, whereas M receives the monitored node V1diode. The nominal application condition is *V*_DD_ = 5.0 V, *I*_BIAS_ = 2 µA, and 25 °C, and the circuit uses 5 V thick-oxide devices in a p-substrate/N-well bulk-CMOS process.

The forward path passes through DIFF, COMPINT, SCHINT, OUTB, and OUT, while DIFFB is the complementary first-stage node. M1–M6 implement the differential transconductor and active load, M21–M22 provide the second gain stage, M30–M33 and M40–M43 form the regenerative network, and M51–M54 restore the output logic level; with the circuit enabled, *V*_P_ > *V*_M_ drives OUT HIGH, whereas *V*_M_ > *V*_P_ drives OUT LOW. The signal-path partitioning is shown in Figure 1.

Table 1 lists the device dimensions taken from the schematic CDL. Here, W and L denote the width and length of one device instance, and m is the number of identical devices connected in parallel. The nominal operating and characterization conditions are listed in Table 2.

### 2.2. Enable and Biasing Circuit

The external bias terminal keeps the original schematic name Ibias1p5u, even though the reference current used throughout this work is $I_{BIAS}\;=\;2\;µA$, so that within the bias network, the diode-connected transistor M4 defines the NMOS mirror-gate voltage, while M3 provides the tail current of the differential pair and M22 sets the current of the second amplification stage; the transistors M11–M16 are instead associated with the enable function and with the clamping of the internal analog nodes. When EN is HIGH, these clamp devices remain inactive and the circuit operates under its normal bias conditions, so that, considering equal channel lengths, similar operating regions, and the corresponding multiplicities, the effective current-scaling ratio between M3, M4, and M22 becomes approximately 2.5:1:1, leading to the following first-order current estimates:

(1) $$I_{tail}\;\approx \;\left(\frac{m_{3}}{m_{4}}\right)I_{BIAS}\;=\;\left(\frac{5}{2}\right)\cdot 2\;µA\;=\;5\;µA;\;\;\;\;\;\;I_{22}\;\approx \;I_{BIAS}\;=\;2\;µA.$$

At zero differential input, the estimated 5 µA tail current is shared approximately equally between M1 and M2, although the transistor-level simulations still account for non-ideal effects such as channel-length modulation, differences in drain voltage, body effect, and process variation. When EN is LOW, M12 suppresses the mirrored NMOS currents, while M14–M16 pull the internal nodes DIFFB, DIFF, and COMPINT toward *V*_DD_. This condition then propagates through SCHINT and OUTB until the final output OUT is driven LOW. The enable and bias circuitry is shown in Figure 2.

### 2.3. Differential Input Stage and Active Load

The transistors M1 and M2 form the NMOS differential pair driven by *V*_P_ and *V*_M_, and M3 supplies their common tail current, while the 1:1 PMOS mirror M5–M6 converts the branch-current difference to the single-ended node DIFF. With *V*_id_ = *V*_P_ − *V*_M_, the two branch currents satisfy *I*_1_ + *I*_2_ ≈ *I*_tail_, and near the balanced operating point their difference is governed by the pair transconductance. The corresponding small-signal current imbalance charges or discharges the extracted capacitance at DIFF according to

(2) $$I_{1}-I_{2}\;\approx \;g_{m}\;V_{id},\;\;\;\;\;\;C_{diff}\cdot dV_{diff}/dt=I_{5}-I_{2}.$$

When *V*_P_ rises relative to *V*_M_, M1 conducts more strongly, DIFFB falls, and M5 sources more current into DIFF, while the opposite sequence occurs when *V*_M_ exceeds *V*_P_. The valid common-mode range is set by the saturation and headroom requirements of M1–M3 and M5–M6, although its exact compact-model limits are not required for the controlled layout comparison.

Therefore, the input pair and its PMOS load are the most direct sources of systematic and random input-referred offset, and their dimensions, orientation, local environment, and interconnect loading must be controlled together; any imbalance between DIFF and DIFFB is amplified by the following stages.

### 2.4. Second Gain Stage

The transistors M21 and M22 implement the second common-source gain stage, with M21 controlled by the voltage at DIFF and M22 acting as an approximately constant 2 µA current sink at the high-impedance node COMPINT. Under small-signal operation, the corresponding stage gain can be approximated as −gm21(ro21∥ro22). An increase in DIFF reduces the conduction of M21 and allows M22 to discharge COMPINT, whereas a decrease in DIFF strengthens M21 and charges the same node. Since both DIFF and COMPINT are high-impedance nodes, the parasitic capacitances extracted around these nodes, together with the associated coupling paths, influence the settling behavior and may also modify the apparent switching point when the signal has a finite slew rate.

### 2.5. Hysteresis Generation

The transistors associated with the SCHINT node implement both the direct inversion and the regenerative feedback required for state-dependent switching. M30 and M40 form the main inverter, while the PMOS stack M31–M33 and the NMOS stack M41–M43 provide the feedback paths selected by OUTB. The node COMPINT drives the direct and intermediate devices, whereas OUTB determines which regenerative branch becomes active. When COMPINT is LOW, the PMOS direct and feedback paths reinforce the HIGH state at SCHINT, while a HIGH level at COMPINT activates the corresponding NMOS paths and reinforces the LOW state. As a result, the effective pull-up and pull-down strengths are not determined only by the instantaneous value of COMPINT, but also by the previously established output state through the regenerative feedback network. The regenerative network is shown in Figure 3.

This is the same positive-feedback principle used in CMOS Schmitt triggers to establish two switching levels and reject slowly varying or noisy inputs [18,19]. Here, however, state dependence is produced by stacked PMOS and NMOS paths rather than by a simple cross-coupled pair. During a rising COMPINT transition, the direct NMOS pull-down must overcome the direct and regenerative pull-up currents; during a falling transition, the direct PMOS pull-up must overcome the direct and regenerative pull-down currents.

These state-dependent current balances produce the internal switching levels *V*_C_^↑^ and *V*_C_^↓^ and the internal hysteresis window *V*_H, C_ = *V*_C_^↑^ − *V*_C_^↓^ > 0, while their exact values remain dependent on compact-model behavior and are evaluated by simulation. The intermediate node voltages, body effect, channel-length modulation, and stack conduction prevent a useful closed-form threshold expression for this topology.

### 2.6. Switching Thresholds and Hysteresis Window

Two threshold domains are used. *V*_C_^↑^ and *V*_C_^↓^ describe the internal SCHINT transitions as COMPINT rises or falls, while *V*_TH_^↑^ and *V*_TH_^↓^ are the input-referred values of *V*_P_ that produce the final stable LOW-to-HIGH and HIGH-to-LOW transitions at OUT for a fixed *V*_M_.

Because the transfer from *V*_id_ to COMPINT is negative, the internal and input-referred switching directions are reversed. Around the operating point, the combined input and second-stage response can be represented locally by *V*_COMPINT_ ≈ *V*_C0_ + *A*_pre_(*V*_P_ − *V*_M_), with *A*_pre_ < 0.

The corresponding input-referred thresholds are *V*_TH_^↑^ = *V*_M_ + (*V*_C_^↓^ − *V*_C0_)/*A*_pre_ and *V*_TH_^↓^ = *V*_M_ + (*V*_C_^↑^ − *V*_C0_)/*A*_pre_. The external hysteresis and switching center are V_H_ = *V*_TH_^↑^ − *V*_TH_^↓^ and *V*_center_ = (*V*_TH_^↑^ + *V*_TH_^↓^)/2, respectively.

These linearized relations describe the local relationship between regeneration, preamplifier gain, offset, and the external switching window, so under finite input slew, the measured crossing also includes settling and propagation.

Figure 4 presents both extraction views. The quasi-static sweep gives *V*_TH_^↑^ = 1.0002 V and *V*_TH_^↓^ = 0.9997 V, corresponding to a 0.534 mV window centered near 1.0000 V, while the triangular-input response gives stable output transitions at 110.4699 µs and 310.6001 µs for *V*_M_ = 1.00 V.

## 3. Symmetry-Oriented Physical-Layout Methodology

This section translates the circuit-level requirements introduced in Section 2 into a set of controlled layout variables, where transistor placement, boundary conditions, isolation, and the routing of critical nodes are intentionally modified from one implementation to another. In this way, the four layouts can be compared not only in terms of the matching benefits obtained through geometrical symmetry but also take into account the parasitic penalties introduced by the physical realization of that symmetry.

### 3.1. Differential-Stage Symmetry Constraints and Comparison Metrics

The M1–M2 differential pair is the most layout-sensitive group because imbalance at its gates, common source, or drain nodes is converted directly into input-referred switching error. The M5–M6 PMOS load is the second matched group; its asymmetry affects both differential-to-single-ended conversion and the loading of DIFF and DIFFB. According to the schematic CDL, every device in these two groups has *W* = 1 µm, *L* = 1 µm, and *m* = 2.

Each schematic instance is implemented as two identical unit devices instead of one wider polygon. This allows the members of each pair to occupy balanced positions. For uncorrelated local mismatch, the area dependence is described by the Pelgrom-type relations [7,8]

(3) $$\begin{array}{c} \sigma ({\Delta V}_{TH})\;\approx \;A_{VTH}/\surd (m\cdot W\cdot L),\\ \sigma (\Delta \beta /\beta )\;\approx \;A_{\beta }/\surd (m\cdot W\cdot L). \end{array}$$

In Equation (3), *A*_VTH_ and Aβ are process-dependent coefficients, W and L are unit-device dimensions, and m is the number of parallel units. In addition to the random mismatch associated with active area, we must also consider equal stress, well proximity, temperature, and interconnect loading. Those deterministic effects are addressed through the placement matrix. In the 2 × 2 abstraction, A and B denote units of M1 and M2, D denotes a dummy, and ∅ denotes an unused position:

(4) $$\begin{array}{c} P\;=\;[p_{\mathrm{ij}}]\;=\;\left[\begin{array}{cc} p_{11} & p_{12}\\ p_{21} & p_{22} \end{array}\right],\\ p_{\mathrm{ij}}\;\in \;\{A,\;B,\;D,\;\emptyset \}. \end{array}$$

For the position vector *r_ij_* = (*x_ij_*, *y_ij_*), common-centroid matching requires the mean positions of the A and B units to coincide [10,11]:

(5) $$\begin{array}{c} {\bar{r}}_{A}\;=\;[\Sigma (i\;\in \;A)\;r_{i}]/N_{A},\\ {\bar{r}}_{B}\;=\;[\Sigma (i\;\in \;B)\;r_{i}]/N_{B},\\ {\bar{r}}_{A}\;=\;{\bar{r}}_{B}. \end{array}$$

Let (*x*0, *y*0) be the center of the active array and let *gx* and *gy* denote the local spatial gradients of a device parameter *q*. With *q(x,y)* = *q*0 + *gx*(*x* − *x*0) + *gy*(*y* − *y*0), the first-order difference between the average A and B parameters is

(6) $$\begin{array}{c} {\Delta q}_{grad}=g_{x}\cdot ({\bar{x}}_{A}-{\bar{x}}_{B})+g_{y}\cdot ({\bar{y}}_{A}-{\bar{y}}_{B}),\\ {\Delta q}_{grad}=0\;\;\;when\;\;\;{\bar{r}}_{A}={\bar{r}}_{B}. \end{array}$$

Centroid coincidence cancels the linear-gradient term in this model, but it does not remove higher-order gradients, local random mismatch, layout-dependent effects, or unequal routing. For the differential stage, the corresponding input-referred contributions can be summarized as

(7) $$\begin{array}{c} V_{OS,eff}\;\approx \;{\Delta V}_{TH}+(V_{OV}/2)\cdot (\Delta \beta /\beta )\\ +V_{OS,grad}+V_{OS,LDE}+V_{OS,par} \end{array}$$

Here, *V*_OV_ is the input-pair overdrive; *ΔV*_TH_ and Δβ/β represent pairwise threshold-voltage and current-factor mismatch; and *V*_OS,grad_, *V*_OS,LDE_, and *V*_OS,par_ denote the input-referred contributions of spatial gradients, layout-dependent effects, and parasitic imbalance. Because these mechanisms cannot be assigned universal priorities, placement is treated as a multi-objective decision. For a candidate matrix P, the comparison vector is

(8) $$\begin{array}{c} F(P)=[{\tilde{E}}_{s}(P),\;{\tilde{E}}_{e}(P),\;R(P),\\ C_{c}(P),\;Ã(P)]. \end{array}$$

In Equation (8), Es describes centroid and orientation error, Ee describes differences in well edge, diffusion edge, contacts, and neighboring geometry, R and Cc represent critical-route resistance and coupling, and A is occupied area. A tile denotes normalization by one fixed, nonzero scale used for all four layouts. No scalar weights are introduced, so C1–C4 are compared component by component. This is important because a lower geometrical error can coexist with larger resistance or coupling, which motivates the compact C4 alternative [10,11].

The unit arrangements presented in Figure 5 are physically implemented in the layouts shown in the later layout examples, where C2 and C3 satisfy the centroid condition defined by Equation (5), while C4 deliberately relaxes this requirement while preserving equal device dimensions, multiplicity, orientation class, dummy boundary, and isolation. Since this modified organization also leads to different routing paths, the associated interconnect parasitics ought to be considered together with the centroid relaxation.

### 3.2. Technology-Dependent Layout Effects and Isolation Strategy

The comparator uses 5 V thick-oxide nfetox5 and pfetox5 devices in a proprietary 130 nm bulk CMOS process with a p-type substrate and N-well isolation. To support reproducibility while respecting process confidentiality, we report the node, device class, well structure, schematic dimensions, extraction content, and electrical test conditions. The comparison concerns one technology and is not a process-node benchmark.

For M5–M6, well-edge proximity can shift threshold voltage and other compact-model parameters when corresponding active regions lie at different distances from the N-well boundary [20,21,22]. Their well-edge distances are constrained to be approximately equal (d_WPE,5_ ≈ d_WPE,6_).

Shallow-trench-isolation stress and length-of-diffusion effects are addressed by matching the source- and drain-side diffusion distances SA and SB, gate orientation, and contact environment for corresponding A and B units.

Only C3 and C4 include edge dummies. These devices give the outer active units a boundary closer to that seen by the inner units and thereby reduce systematic end effects [23]. They do not contribute to electrical multiplicity and are connected according to the design rules. Because C3 introduces both dummies and continuous guard rings relative to C2, the C2–C3 result represents their combined effect; the two mechanisms are not isolated by the present comparison.

Bulk CMOS contains vertical PNP and lateral NPN parasitic paths that can form a PNPN structure. To collect injected carriers and reduce local well and substrate voltage excursions, C3 and C4 use a continuous N+ ring in the N-well tied to *V*_DD_ and a continuous P+ ring in the substrate tied to *V*_SS_ [24,25]. These rings reduce latch-up susceptibility but do not eliminate the intrinsic parasitic bipolar paths. C1 and C2 retain only the well and substrate contacts required by the design rules. The relevant well and guard-ring geometry is illustrated in Figure 6.

These collection rings control the local well and substrate potential and provide electrical isolation; they are not part of the M1–M2 matching matrix. Their perimeter and spacing are treated as a separate physical cost and included in the measured C3/C4 footprint.

### 3.3. Construction of the Four Layout Variants

All four layouts are instantiated from the same comparator schematic. Device models, dimensions, multiplicities, connectivity, pin names, and bias interface are unchanged, and each implementation passes DRC and LVS before extraction. Thus, the electrical netlist is identical and only physical realization changes.

C1 is the unconstrained baseline. Its schematic devices were placed with the Cadence Virtuoso placement facility, without manual common-centroid, interdigitation, or pair-symmetry constraints. It is a tool-generated device placement rather than a fully automatic layout-synthesis result, a distinction that matters when it is compared with layout-aware automation methods [12,16,17].

C2 is manually constrained for symmetry. The two units of M1 and the two units of M2 form a 2 × 2 AB/BA common-centroid pattern. M5–M6 and the remaining corresponding devices use consistent orientation and local environment. C2 contains neither edge dummies nor continuous N+/P+ guard rings. Its differential core is

(9) $$P_{C2}=\left[\begin{array}{cc} A & B\\ B & A \end{array}\right].$$

C3 keeps the M1–M2 common centroid and the matched M5–M6 organization, then adds the edge dummies and continuous N+/P+ guard rings described in Section 3.2. Since C2 and C3 differ by both additions, their comparison measures a combined boundary-and-isolation change rather than the effect of either feature alone.

C4 retains the same edge dummies, continuous guard rings, and matched M5–M6 organization as C3. Its M1–M2 unit arrangement changes from AB/BA common centroid to adjacent A and B columns (AB/AB by rows). Moving these units also moves their terminals and changes the available route lengths and proximities; the input gates, common source, and sensitive drain connections therefore follow a different routing geometry:

(10) $$P_{C4}\;=\;\left[\begin{array}{cc} A & B\\ A & B \end{array}\right].$$

The C4 matrix in Equation (10) relaxes first-order centroid cancellation. C3 and C4 have the same dummy boundary, guard rings, and footprint, but their M1–M2 positions and resulting routes differ. Keeping the exact route geometries fixed when terminal coordinates change is generally infeasible. We therefore interpret their measured electrical difference as the combined response of device placement and interconnect routing; this experiment cannot assign separate causal contributions to these changes.

Table 3 lists the controlled differences.

Figure 7 establishes two useful controls. First, manual matching does not necessarily enlarge the cell: C2 has a shallower outline and occupies 30.68% less area than C1. Second, C3 and C4 have the same outline and corner void. Their differences cannot be attributed to occupied area, dummy count, or guard-ring perimeter; they are associated primarily with the changed M1–M2 organization and the routing produced by it.

For the L-shaped layouts C3 and C4, occupied area is calculated from the measured bounding box after subtracting the corner void reported in Table 4.

The normalized area change from reference layout *j* to layout *i* is

(11) $$\begin{array}{c} r_{A}(i,j)=A_{occ}(i)/A_{occ}(j),\\ \Delta A(i\leftarrow j)\;[\%]=100\cdot [r_{A}(i,j)-1]. \end{array}$$

Table 4 gives the resulting dimensions. The C2 void occupies 23.64 µm^2^, or 5.23% of its bounding box, whereas the 80.80 µm^2^ C3/C4 void represents 8.61%. Relative to C2, the protected C3/C4 footprint increases by 100.12%. This is the combined cost of dummies, continuous guard rings, isolation spacing, and the associated floorplan; it is not attributed to dummies alone.

The close-up views in Figure 8 show that C4 preserves the same two-unit devices for both M1 and M2, together with the dummy field, guard structure, and the matched organization of M5–M6 already used in C3. However, the AB/BA centroid arrangement is replaced by adjacent A and B columns, which modifies both the degree of first-order centroid cancellation and the resulting interconnect geometry; consequently, the C3–C4 comparison reflects the overall trade-off between the matching advantage provided by the centroid structure and the routing parasitics introduced by each physical organization.

### 3.4. Post-Layout Extraction and Comparative Evaluation Methodology

All four layouts were implemented in Cadence Virtuoso Studio IC23.1-64b.ISR15.37 and subsequently extracted with Calibre Interactive PEX v2024.2_18.9 using the same xrc.rs. Each layout was verified as DRC clean and LVS equivalent before applying transistor level accuracy 200 extraction with R+C+CC enabled and SPEF used as the output format. The resulting extracted networks include interconnect resistance, capacitance referenced to ground, and explicit pairwise coupling capacitances, while the process setup, extraction options, and nominal comparison conditions were kept unchanged throughout the study. Under these controlled conditions, the physical layout remains the intended comparison variable, and the reported differences are those captured by the same extraction models and settings [16,17,21].

For each critical node *i* ∈ {P, M, DIFF, DIFFB, COMPINT, SCHINT, OUTB, and OUT}, the extracted ground capacitance and all pairwise coupling terms are summed to obtain the reported total node capacitance:

(12) $$C_{total}(i)\;=\;C_{gnd}(i)\;+\;\Sigma (j\;\neq \;i)\;C_{cpl}(i,j).$$

The principal physical metrics are occupied area, total critical-node capacitance, input-capacitance imbalance, and the distributed resistance balance of the P and M routes. In addition to these primary quantities, individual coupling terms, other node resistances, critical-route length, and via count remain auxiliary diagnostics, so they are used to explain an electrical difference where needed, but they are not presented as independent optimization targets for every layout.

The common testbench uses *V*_M_ = 1.00 V, *I*_BIAS_ = 2 µA, *C*_L_ = 10 fF, and T = 25 °C. The main characterization supply is *V*_DD_ = 5.0 V, and a second evaluation is performed at 1.8 V. A 1.8 V supply was selected above the minimum supported limit, while 5.0 V represents the nominal supply and 1.0 V was used as the fixed threshold-reference input.

The PWL stimulus applied to *V*_P_ is 0.950 V from 0 to 10 µs, rises linearly to 1.050 V at 210 µs, returns linearly to 0.950 V at 410 µs, and remains there until 420 µs. The same waveform excites both switching directions. Temperature sweeps use the same PEX netlist, bias, load, waveform, and numerical criteria at every point. The verification flow and evaluation plan are summarized in Figure 9 and Table 5, respectively.

Section 4 reports the finite-slew, temperature, process-corner, and statistical controls together with their results; within each comparison, all layouts use the same numerical settings and measurement rules.

## 4. Results

Case C0, the schematic reference, and the four extracted layouts C1–C4 were evaluated with identical device dimensions, bias, 10 fF output load, input waveform, and crossing criterion. The results proceed from the extracted parasitics to nominal switching, temperature and supply robustness, and statistical behavior.

### 4.1. Extracted Parasitic Comparison of the Four Layouts

Calibre xRC v2024.2_18.9 generated the four final post-layout netlists with transistor-level R+C+CC extraction and no inductance. In each SPEF file, a one-node *CAP entry represents a ground-referenced contribution, whereas a two-node entry represents an explicit coupling capacitance. The declared units are 1 pF and 1 Ω; all reported capacitances were converted to femtofarads. Table 6a reports the D_NET total capacitances of the critical internal nodes according to Equation (12), while Table 6b separates total input capacitance, explicit input coupling, and distributed input-route resistance.

For each external input, the incident coupling sum is obtained from all explicit two-node capacitors connected to that net. The direct P–M term is also reported separately, while the difference between the two coupling environments is defined by Equations (13)–(15). Coupling entries repeated in the partner D_NET section were not counted twice.

(13) $${CC}_{\Sigma }(P)=\sum_{j\;\neq \;P}\;CC(P,j)$$

(14) $${CC}_{\Sigma }(M)=\sum_{j\;\neq \;M}\;CC(M,j)$$

(15) $$\Delta {CC}_{in}=\left|{CC}_{\Sigma }(P)-{CC}_{\Sigma }(M)\right|$$

Table 6a shows that, relative to C3, C4 lowers the total capacitance of all six reported internal nodes by approximately 4.5–24.7%. Table 6b separates direct mutual coupling from coupling imbalance. C2 has the largest direct P–M coupling, 1.1036 fF, but its incident-coupling imbalance is only 0.0562 fF; C1 has a smaller direct P–M term of 0.3227 fF and the largest imbalance, 4.6076 fF. CC(P,M) by itself is an incomplete descriptor of input-route symmetry. C4 combines the lowest direct P–M coupling, 0.0962 fF, with the smallest coupling imbalance, 0.0025 fF, corresponding to reductions of 81.7% and 99.2% relative to C3. Moreover, its P and M resistance sums agree within less than 0.001 Ω, and their mean is 30.3% below that of C3. These values place C4 closest to electrical balance at the extracted input routes. A network-level interpretation is still required for the later ripple difference, because no single coupling capacitor explains it.

### 4.2. Nominal Post-Layout Switching and Output Integrity

Nominal transients were evaluated at 25 °C for *V*_DD_ = 1.8 V and 5.0 V. The 1.8 V case is a low-voltage operating point above approximately 1.6 V technology minimum. *V*_M_ was fixed at 1.000 V, while *V*_P_ followed the 0.950-1.050-0.950 V triangular waveform defined in Section 3. Rising and falling events are taken as the last 50% output crossings after which OUT remains in the new logic state. With *S*_P_ = 0.5 mV/µs, the corresponding input voltages are

(16) $$V_{P,sw}^{↑}=0.950V+S_{P}(t_{up,last}-10\mu s)$$

(17) $$V_{P,sw}^{↓}=1.050V-S_{P}(t_{down,last}-210\mu s)$$

The apparent finite-slew switching window and its displacement from the fixed *V*_M_ input are then

(18) $$V_{H,FS}=V_{P,sw}^{↑}-V_{P,sw}^{↓},$$

(19) $${\Delta V}_{center}=\frac{V_{P,sw}^{↑}+V_{P,sw}^{↓}}{2}-V_{M}$$

Equations (16)–(19) are obtained from a finite-slope transient waveform. Accordingly, *V*_H,FS_ is an apparent switching window that includes dynamic settling and is not presented as a purely static DC hysteresis value. The nominal switching metrics are reported in Table 7.

At 5.0 V and 25 °C, the finite-slew switching window is 0.845 mV for schematic C0 and 1.549, 1.087, 0.986, and 1.027 mV for layouts C1–C4, respectively. C3 has the narrowest window among the extracted layouts. The corresponding absolute center displacements are 291.4, 231.9, 113.1, and 110.7 µV for C1–C4. C4 has the smallest nominal value in this specific test, but the 2.4 µV separation from C3 does not establish greater mismatch robustness.

Using the last stable crossing prevents the early C1 pulses in Figure 10 from being counted as functional transitions. C2–C4 show no corresponding pre-switching pulses at any of the six temperature and supply combinations.

At 5.0 V and 25 °C, the extracted layouts span more than three orders of magnitude in settled high-state ripple: C1 gives 2.001 mV, C2 gives 315.758 mV, C3 gives 18.693 mV, and C4 gives only 0.275 mV. Relative to C1, C2, and C3, the C4 organization reduces this metric by 86.269%, 99.913%, and 98.530%, respectively. The particularly large C2 value also shows why placement regularity cannot be interpreted independently of the interconnect network, since strict matching alone does not prevent a routing- or coupling-dominated output disturbance. C0 is not included in Figure 11 because it is a schematic reference and its 0.0001 mV value does not contain extracted-layout parasitics.

### 4.3. Parasitic Mechanism Behind the C2 Output Ripple

C2 provides a useful counterexample because its geometrically regular organization produces closely matched input capacitance totals, |ΔCtot| = 0.0564 fF, but the same layout gives the largest simulated high-state ripple. The broader extracted network explains why the input imbalance alone is insufficient: relative to C4, C2 increases the direct P–M coupling from 0.0962 fF to 1.1036 fF and the mean distributed input-route resistance from 25.908 Ω to 39.7335 Ω. Along the amplified signal path, the combined DIFF/DIFFB-to-COMPINT coupling is 1.7101 fF for C2 and 0.5966 fF for C4, while the COMPINT-to-OUTB coupling rises from 0.1445 fF to 0.8518 fF. In addition to these coupling differences, the total SCHINT capacitance is 5.075 fF for C2 and 3.395 fF for C4.

A supplementary extraction performed on the same C2 geometry and with the same testbench further supports this interpretation, since reducing the parasitic model from R+C+CC to R+C lowers the settled high-state ripple below 0.001 mV, whereas the complete coupling model preserves the approximately 0.3 V disturbance, indicating that explicit inter-node coupling, rather than distributed resistance and ground-referenced capacitance alone, is the main mechanism responsible for triggering the regenerative disturbance observed at the output.

The extracted coupling terms and the selective capacitance-zeroing tests support a stronger interpretation of the C2 disturbance. In the original extracted circuit, output ripple is 315.758 mVpp at 25 °C and 5 V. For the sensitivity tests, all extracted capacitors incident on the named node(s) were set to zero in separate copies of the same netlist; the circuit devices and other parasitic components were retained. The combined DIFF and DIFFB intervention lowers ripple to about 56.9 mVpp. These node-group experiments localize a large contribution to the differential-stage coupling network; they do not isolate or rank individual DIFF–input capacitor pairs.

DIFF and DIFFB are switching nodes near the two inputs. Opposing transitions can increase transient capacitive current into a susceptible input, while the downstream gain and feedback path can magnify the resulting disturbance. This describes a circuit-consistent mechanism; the tests below quantify node-group sensitivity rather than the direction or magnitude of each individual coupling path. The sensitivity results are listed in Table 8.

The ripple is evaluated as peakToPeak(clip(V(OUT), 180 µs, 240 µs)) in millivolts, independently of the last-stable-crossing rule used for transition times. Extending the transient stop time from 430 to 600 µs changes the same-window measurement from 313.647 to 314.291 mVpp (0.21%). Thus, the observed large ripple persists in a fixed interval well before either simulation ends. The 315.758 mVpp result is retained as the original reported value; the reruns reproduce it within 1%.

### 4.4. Temperature and Supply Robustness

The same PEX experiment was repeated at −40 °C, 25 °C, and 125 °C for both supply voltages. Figure 12 and Figure 13 compare C0–C4 at the nominal process corner; the separate mismatch experiment in Section 4.6 uses the nominal 5.0 V supply.

Figure 12 shows that temperature shifts both switching thresholds upward in every case, with a magnitude that depends on layout and supply. At 25 °C, the C3 and C4 rising thresholds differ by 17.9 µV and their falling thresholds by 22.7 µV. These small nominal differences cannot, on their own, explain the much larger separation in output ripple.

Figure 13 separates finite-slew switching-window width from signed center displacement. The schematic reference C0 has the narrowest window, and C3 has the narrowest window among extracted layouts at every plotted temperature and supply. C4 has the smallest absolute center displacement in this deterministic grid. At 5.0 V, its values improve on C3 by 6.9%, 2.1%, and 10.7% at −40 °C, 25 °C, and 125 °C, respectively. These deterministic comparisons do not establish lower mismatch-induced center dispersion.

### 4.5. Process-Corner Robustness

To distinguish deterministic process-corner movement from local mismatch, C0, C3, and C4 were evaluated at 25 °C across SS, SF, TT, FS, and FF for both 1.8 V and 5.0 V.

Cases C1 and C2 are not included in this figure because only nominal-corner results are available for them. The switching window and signed center displacement were calculated from the same last-stable crossings used in Section 4.4, while the settled high-state ripple was evaluated over the unchanged observation interval.

Figure 14 shows the process-corner comparison at 25 °C. For C3, the finite-slew switching window spans 0.649–0.749 mV at 1.8 V and 0.956–1.037 mV at 5.0 V; the corresponding C4 ranges are 0.654–0.764 mV and 0.982–1.092 mV. C4 has smaller settled high-state ripple in this 25 °C sweep, while the switching metrics remain similar. Figure 14 includes the schematic C0 reference; the additional temperature sweeps below retain C0 alongside C3 and C4.

The comparison was extended to a mixed PVT grid for the schematic C0 reference and extracted C3 and C4. Each case was evaluated at the five available process corners (SS, SF, nominal/TT, FS, and FF), at −40, 25, and 125 °C, and at 1.8 and 5.0 V: 30 matched conditions per case. Figure 15A,B report the −40 and 125 °C results. The same triangular stimulus, last-stable crossing rule, and settled high-state ripple metric were used throughout.

Across the added −40 and 125 °C corners, C4’s settled high-state ripple ranges from 0.0678 to 0.0990 mV at 1.8 V and from 0.2269 to 0.2936 mV at 5.0 V; the corresponding C3 ranges are 4.773–6.761 mV and 15.623–20.551 mV. At every matched PVT combination in the supplied grid, C4 has lower ripple than C3, while C3 has the narrower apparent finite-slew switching window among the extracted layouts. Schematic C0 provides a separate low-ripple reference. The signed center displacement shifts with temperature, and these deterministic runs do not establish a mismatch-dispersion advantage for either layout.

### 4.6. Monte Carlo Statistical Robustness

Mismatch Monte Carlo simulations were repeated for C3 and C4 at the nominal process corner, 25 °C, and the nominal *V*_DD_ = 5.0 V. Both runs used the Standard Monte Carlo method with Mismatch variation, 200 low-discrepancy points, seed 12345, first point 1, *C*_L_ = 10 fF, a 430 µs transient stop time, and conservative simulator accuracy. The runs used the PDK matching section; no additional spatial-correlation or position-dependent mismatch analysis was configured or evaluated. The PDK model coefficients and any embedded correlation are proprietary and were not independently verified. Accordingly, these results characterize the variation returned by the selected mismatch configuration and do not establish immunity to spatial gradients.

For each sample, *t*_up,last_ is the last rising crossing of OUT at 2.5 V within 10–210 µs, *t*_down,last_ is the last falling crossing at 2.5 V within 210–410 µs, and *V*_ripple,pp_ is the peak-to-peak output voltage from 180 to 240 µs. These definitions and measurement windows are identical for C3 and C4.

Equations (16)–(19) were applied to each sample with *V*_P,0_ = 0.950 V, *V*_P,1_ = 1.050 V, t_0_ = 10 µs, t_1_ = 210 µs, a rising input slew of 0.5 mV/µs, and *V*_M_ = 1.000 V. Sample-by-sample conversion preserves the covariance between thresholds. The resulting hysteresis is an apparent finite-slew window rather than a quasi-static DC value. The resulting Monte Carlo timing and ripple distributions are shown in Figure 16.

The timing distributions are similar. For C3, the mean rising and falling times are 111.282 µs and 310.678 µs, with sample standard deviations of 21.482 µs and 21.437 µs. For C4, the corresponding means are 111.280 µs and 310.780 µs, and the deviations are 21.672 µs and 21.648 µs. These close dispersions do not establish a practical timing robustness advantage for either layout.

The output-ripple distributions differ strongly: C3 has a sample mean of 18.6888 mVpp and a sample standard deviation of 0.1060 mV, whereas C4 gives 0.2743 mVpp and 0.0021 mV. The C4 mean is 98.53% lower in this experiment. All observed C4 values (0.2674–0.2794 mVpp) are below the observed C3 range (18.4332–18.9560 mVpp).

The voltage-domain distributions obtained from the same 200 mismatch samples are shown in Figure 17.

C3 has sample mean rising and falling thresholds of 1000.6409 mV and 999.6611 mV, with standard deviations of 10.7408 mV and 10.7186 mV. The corresponding C4 means are 1000.6402 mV and 999.6102 mV, with standard deviations of 10.8362 mV and 10.8239 mV. Threshold dispersion differs by about 1% between the two layouts.

Within each layout, rising and falling thresholds are highly correlated (ρ > 0.9999): mismatch mainly translates the switching window. C3 has an apparent finite-slew window of 0.9799 ± 0.0434 mV (sample mean ± standard deviation), compared with 1.0301 ± 0.0284 mV for C4. The signed center-displacement means are +0.1510 mV and +0.1252 mV, respectively, while their standard deviations are 10.7297 mV and 10.8301 mV. The approximately 25.8 µV difference between center means is small relative to either spread; it does not establish a mismatch-centering advantage.

All 200 samples produced the three reported outputs for each layout. The sample size supports descriptive means, standard deviations, and observed ranges for this nominal mismatch condition. No specification limits or rare-event sampling were used, so the experiment does not estimate production yield or extreme-tail probabilities.

### 4.7. Discussion

The preferred physical implementation depends on the measured specification. At the tested nominal conditions, C3 gives the narrowest finite-slew switching window among extracted layouts, whereas C4 gives the lowest settled high-state ripple. C1 exhibits pre-switching chatter, and C2 has a large nominal ripple despite its regular geometry. The mixed PVT grid shows lower ripple for C4 at each simulated combination, but the 5.0 V mismatch experiment does not show a meaningful advantage in threshold or center dispersion. Thus, selecting C4 for ripple does not imply general statistical superiority.

Common-centroid placement and edge dummies remain useful for limiting spatial and boundary effects [10,11]. The electrical differences measured here also depend on the interconnect produced by the layout. In C3 and C4, terminal relocation changes route lengths and proximities, so the experiment tests complete placement-and-routing solutions. The separate process-corner and mismatch experiments probe different variation sources and neither is a production-yield study.

The same evaluation principle may be useful in other comparator topologies, current mirrors, oscillators, and analog blocks. Relevant nets should first be classified by how susceptible they are to coupled disturbance and by how strongly their voltage or current transitions can disturb neighboring nets. The applied input amplitude and slew rate affect that classification in a comparator. A layout may then relax a geometric matching constraint if a lower extracted coupling error outweighs the added mismatch error. This is a design hypothesis for other architectures, not an experimentally verified result of the present single-comparator study; each extension requires its own layout extraction and circuit simulation.

## 5. Conclusions

Four DRC-clean, LVS-equivalent physical layouts of the same 130 nm CMOS hysteresis comparator were compared under a fixed schematic, stimulus, bias, load, and R+C+CC extraction flow. The study measured input and internal-node parasitics, finite-slew switching thresholds and window, switching-center displacement, and settled high-state output ripple. Schematic C0 served as a reference alongside the four extracted layouts C1–C4.

At 5.0 V and 25 °C, the strictly common-centroid C2 layout produced 315.758 mVpp output ripple despite balanced input capacitances. The measured waveform, fixed-window reruns, and node-group capacitance interventions support a substantial contribution from the DIFF/DIFFB-associated coupling network; they do not identify a single dominant capacitor. Among the extracted layouts, C3 gave the narrowest nominal finite-slew switching window, whereas C4 gave the lowest nominal output ripple.

In the 30 matched combinations of process corner, supply, and temperature simulated for C0, C3, and C4, C4 had lower high-state ripple than C3. At the nominal 5.0 V supply, 200 mismatch-only samples per layout yielded mean ripple values of 18.6888 mVpp for C3 and 0.2743 mVpp for C4. Their threshold and switching-center dispersions were similar, so these samples do not establish a general mismatch-robustness or yield advantage for either layout.

The physical comparisons concern complete implementations: C2–C3 changes dummies and guard rings together, while C3–C4 changes placement and routing together. For other comparator architectures, current mirrors, or oscillators, the potential value of relaxing a geometric matching constraint depends on the sensitivity of nearby nets and the strength of their switching disturbances. Those applications require their own extracted-layout simulations and, ultimately, silicon validation.

## Figures and Tables

**Figure 1:**
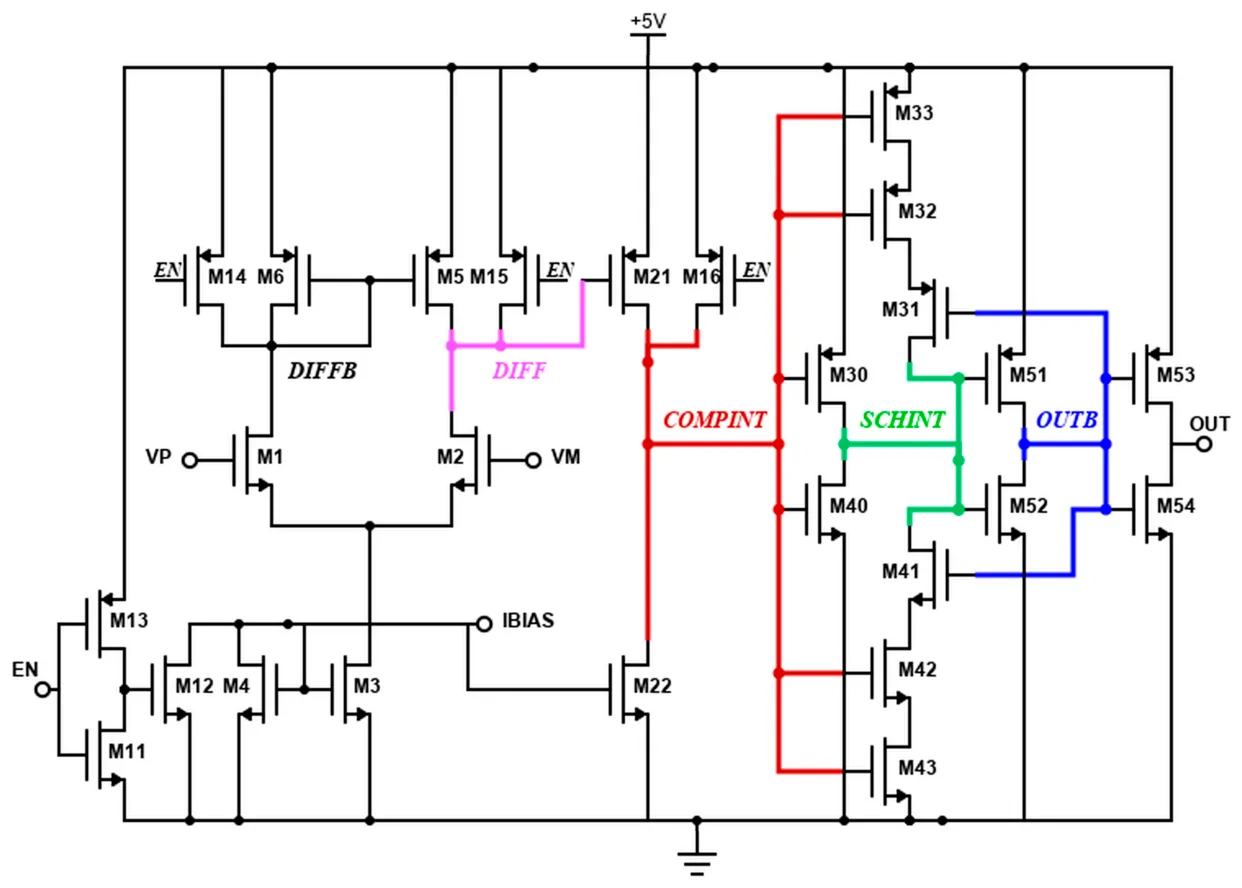
Transistor-level architecture and signal-path partitioning of the investigated CMOS hysteresis comparator. *V*_P_ and *V*_M_ denote the voltages at input terminals P and M; DIFFB, DIFF, COMPINT, SCHINT, OUTB, EN, and OUT identify the remaining labeled nodes and signals. The EN-controlled devices establish the enabled and disabled states.

**Figure 2:**
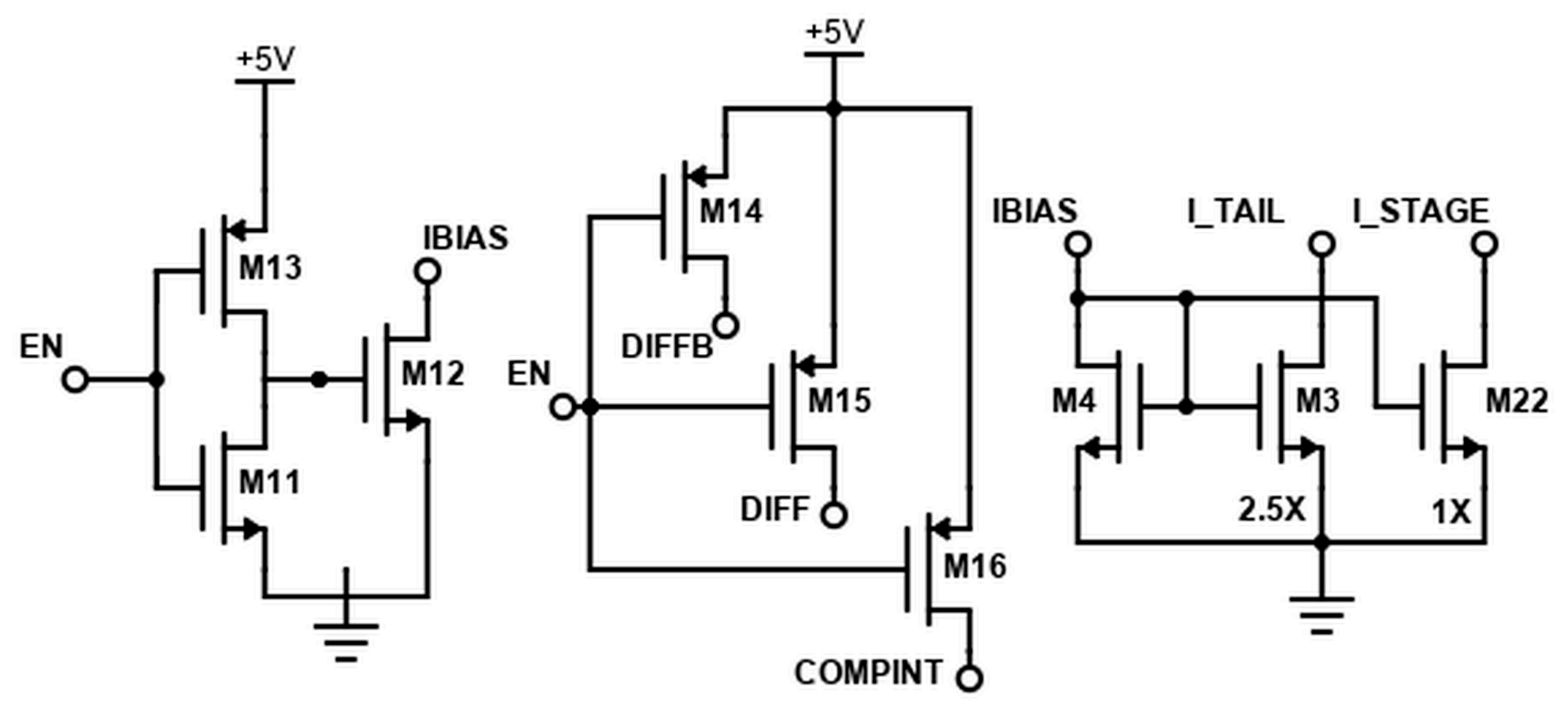
Enable, clamp, and bias-current generation circuitry: enable inverter and bias clamp (**left**), disabled-state PMOS pull-up network (**center**), and current-mirror branches generating *I_TAIL_* and *I_STAGE_* (**right**).

**Figure 3:**
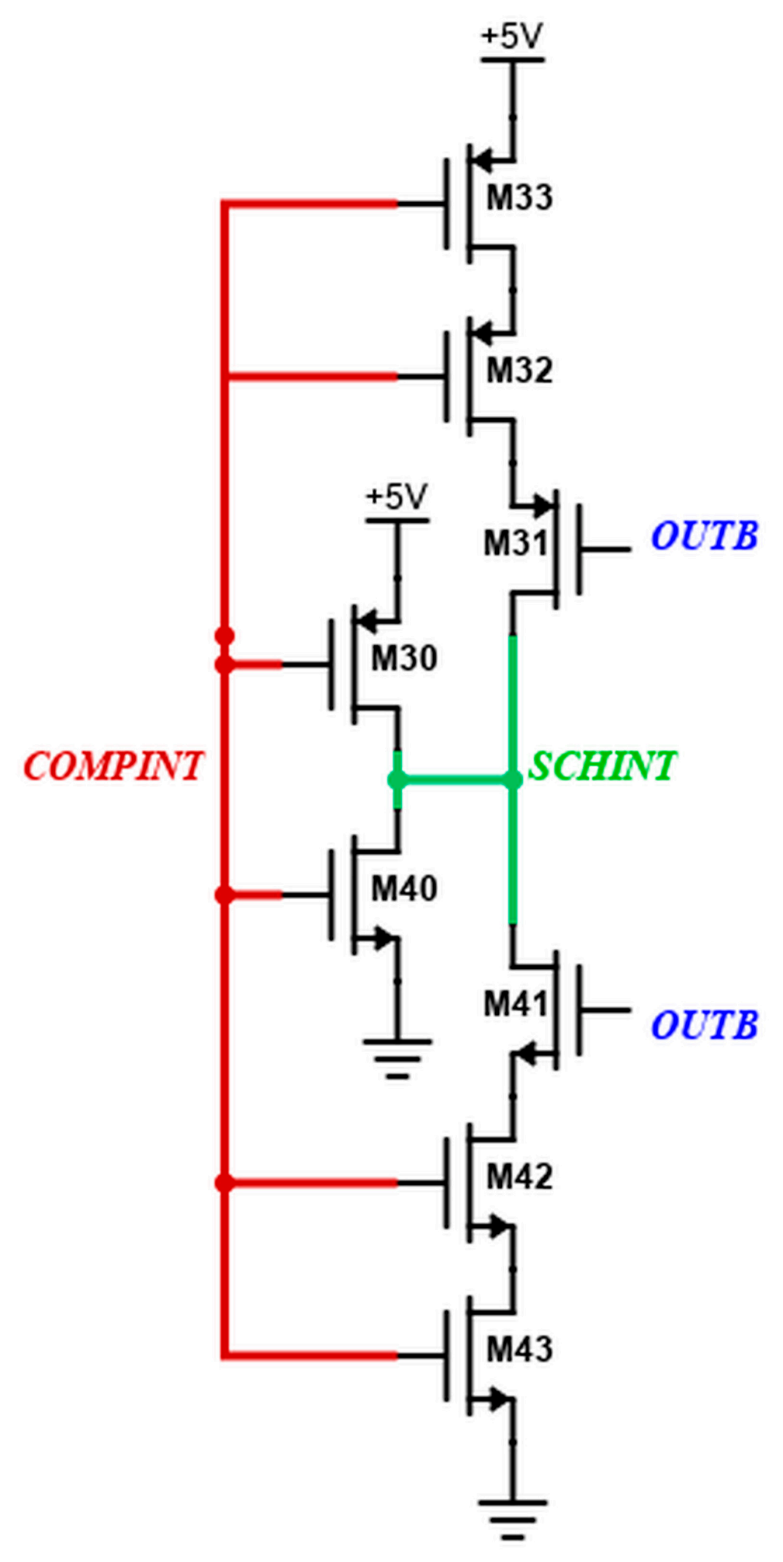
Regenerative hysteresis network and state-dependent feedback paths. The direct inverter is controlled by *COMPINT*, whereas *OUTB* selects the upper or lower regenerative branch. Red identifies COMPINT, blue identifies OUTB, and green identifies SCHINT.

**Figure 4:**
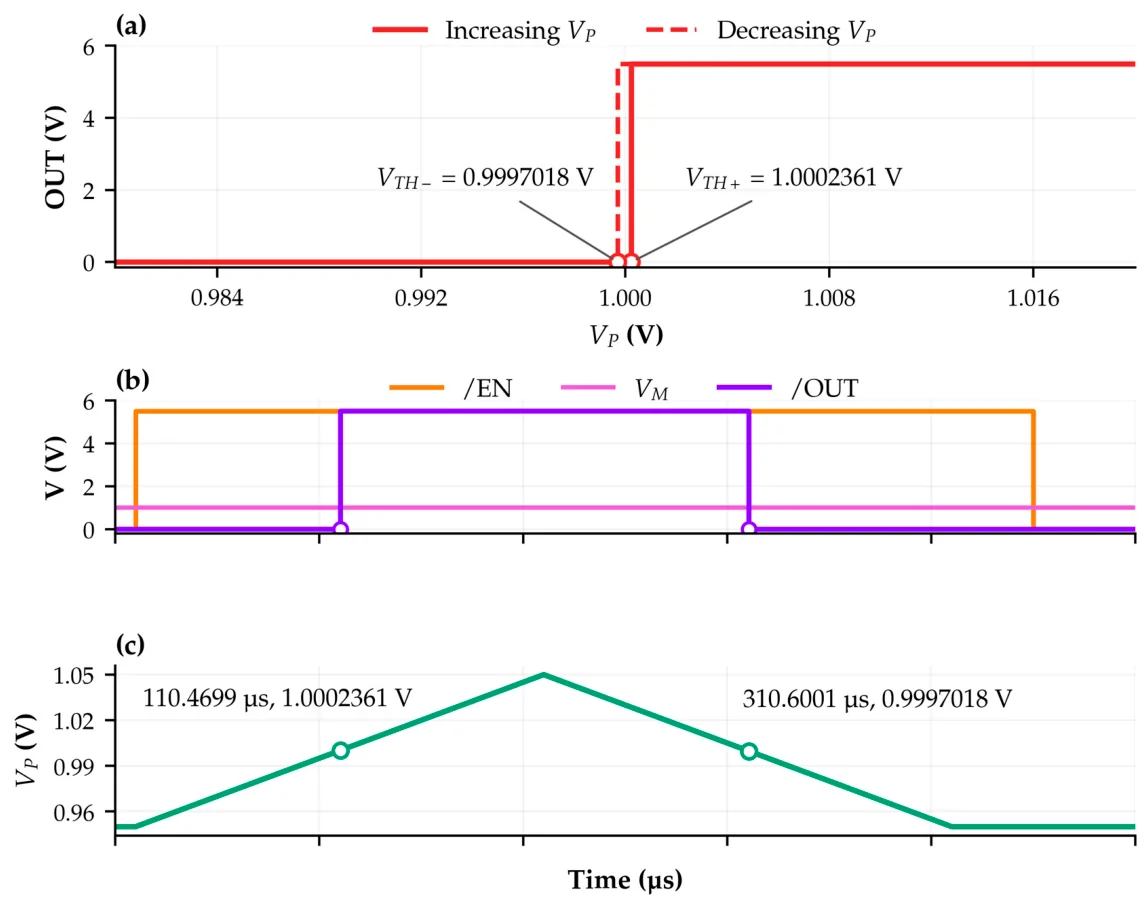
Input-referred hysteresis characterization under quasi-static and transient excitation: (**a**) *OUT* versus *V*_P_ for increasing and decreasing input sweeps; (**b**) /EN, *V*_M_, and /OUT during the transient test; and (**c**) the triangular *V*_P_ waveform at constant *V*_M_, with switching markers.

**Figure 5:**
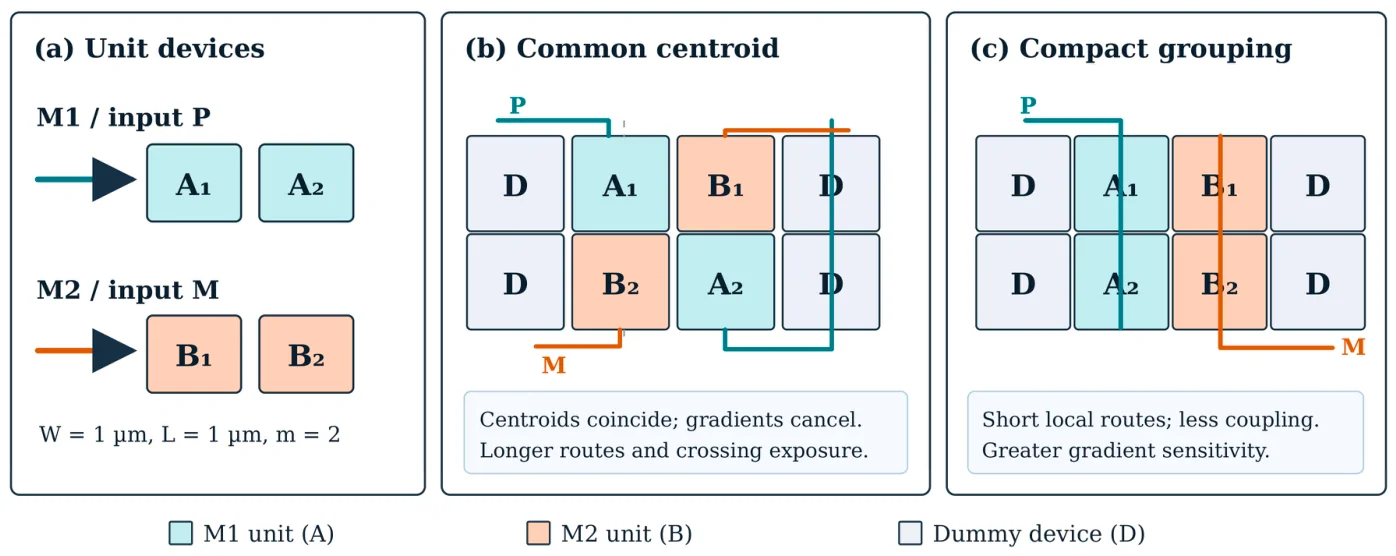
Matrix-based abstraction of the M1–M2 differential pair and comparison between common-centroid and AB/AB column-grouped parasitic-oriented placement. The edge dummies shown around the two example patterns illustrate the common boundary used when C3 and C4 are compared; C2 does not contain these dummies.

**Figure 6:**
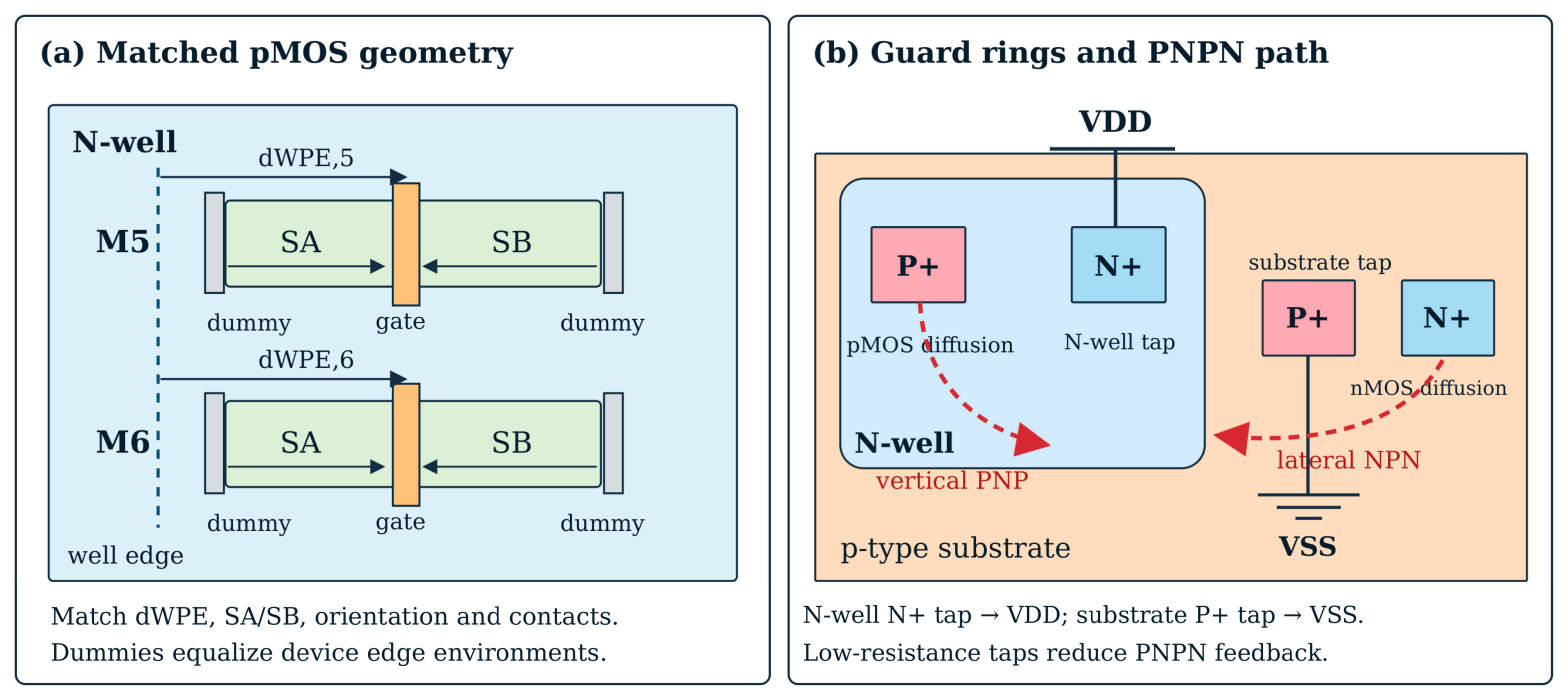
Conceptual representation of layout-dependent geometry and isolation in bulk CMOS: (**a**) well-edge distance dWPE, diffusion-edge distances SA and SB, and edge dummies for a matched PMOS pair; (**b**) simplified p-substrate/N-well cross-section showing the N+ and P+ guard rings and the parasitic PNPN feedback path. The drawing does not reproduce the proprietary process cross-section.

**Figure 7:**
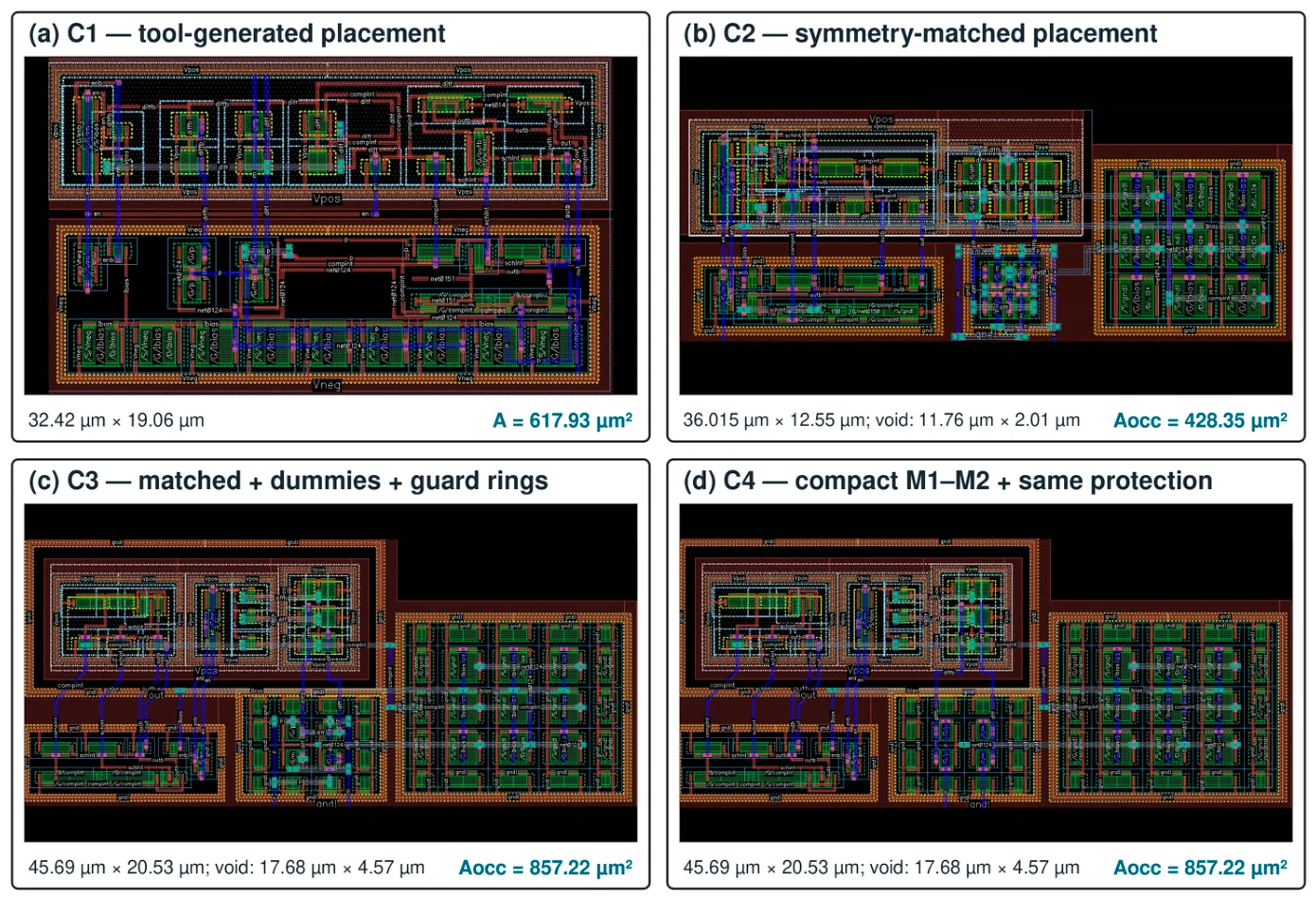
Full-layout views of (**a**) C1, (**b**) C2, (**c**) C3, and (**d**) C4. Area measurements are reported in Table 4.

**Figure 8:**
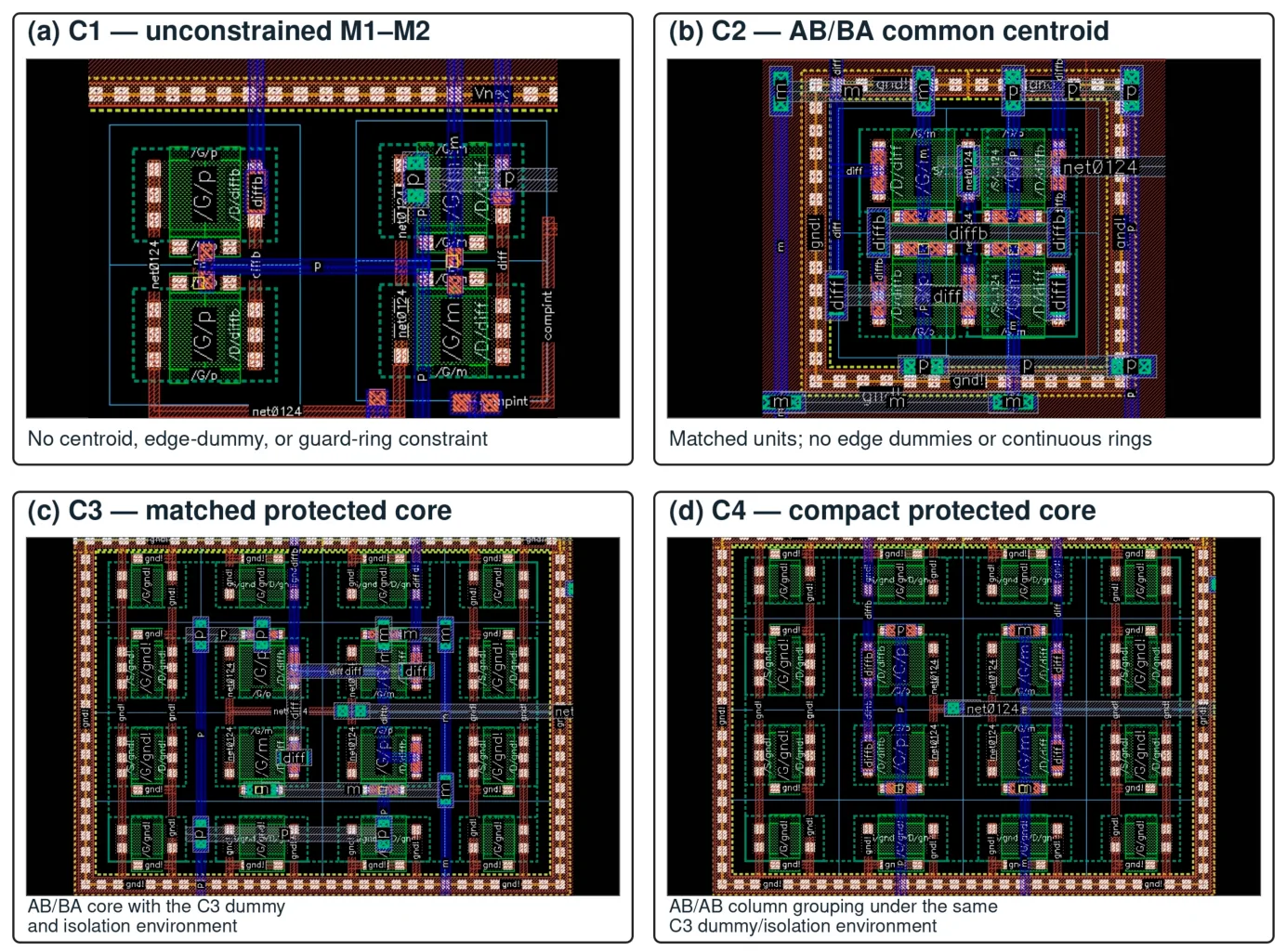
Differential-stage layout details for (**a**) C1, (**b**) C2, (**c**) C3, and (**d**) C4. The four panels show the changes in placement, dummies, and surrounding isolation.

**Figure 9:**
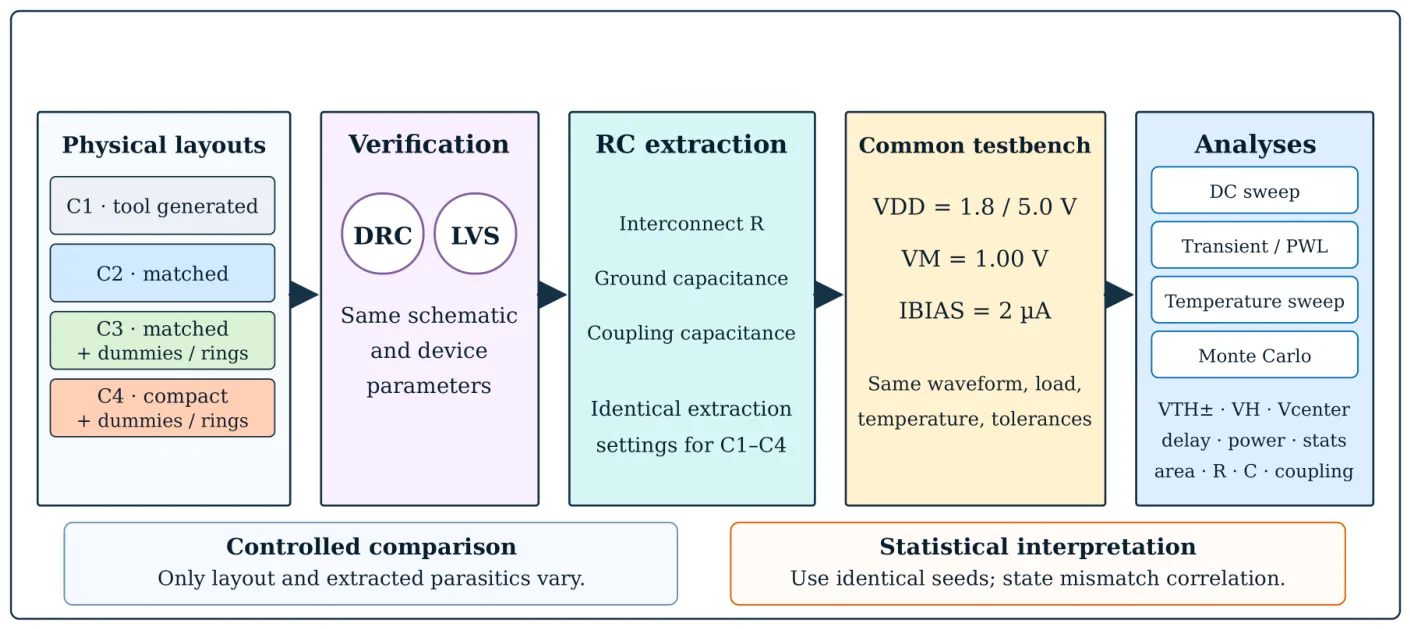
Common verification, parasitic extraction, and simulation flow applied to C1–C4 under identical settings, stimuli, and numerical measurement criteria.

**Figure 10:**
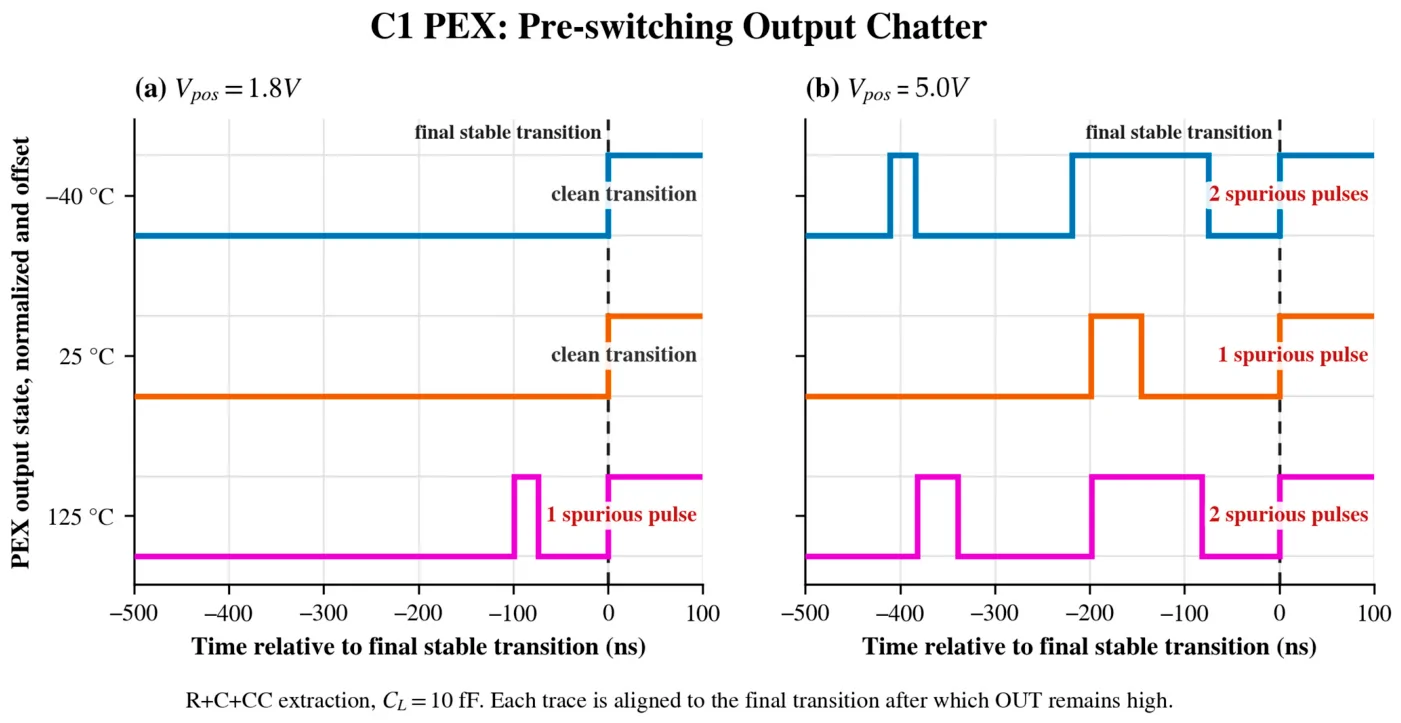
C1 pre-switching output chatter under R+C+CC extraction at (**a**) *V*_DD_ = 1.8 V and (**b**) *V*_DD_ = 5.0 V. Each trace is aligned to the final stable rising transition. C1 is clean at −40 °C and 25 °C for 1.8 V, produces one spurious pulse at 125 °C for 1.8 V, and produces one or two spurious pulses at all three temperatures for 5.0 V.

**Figure 11:**
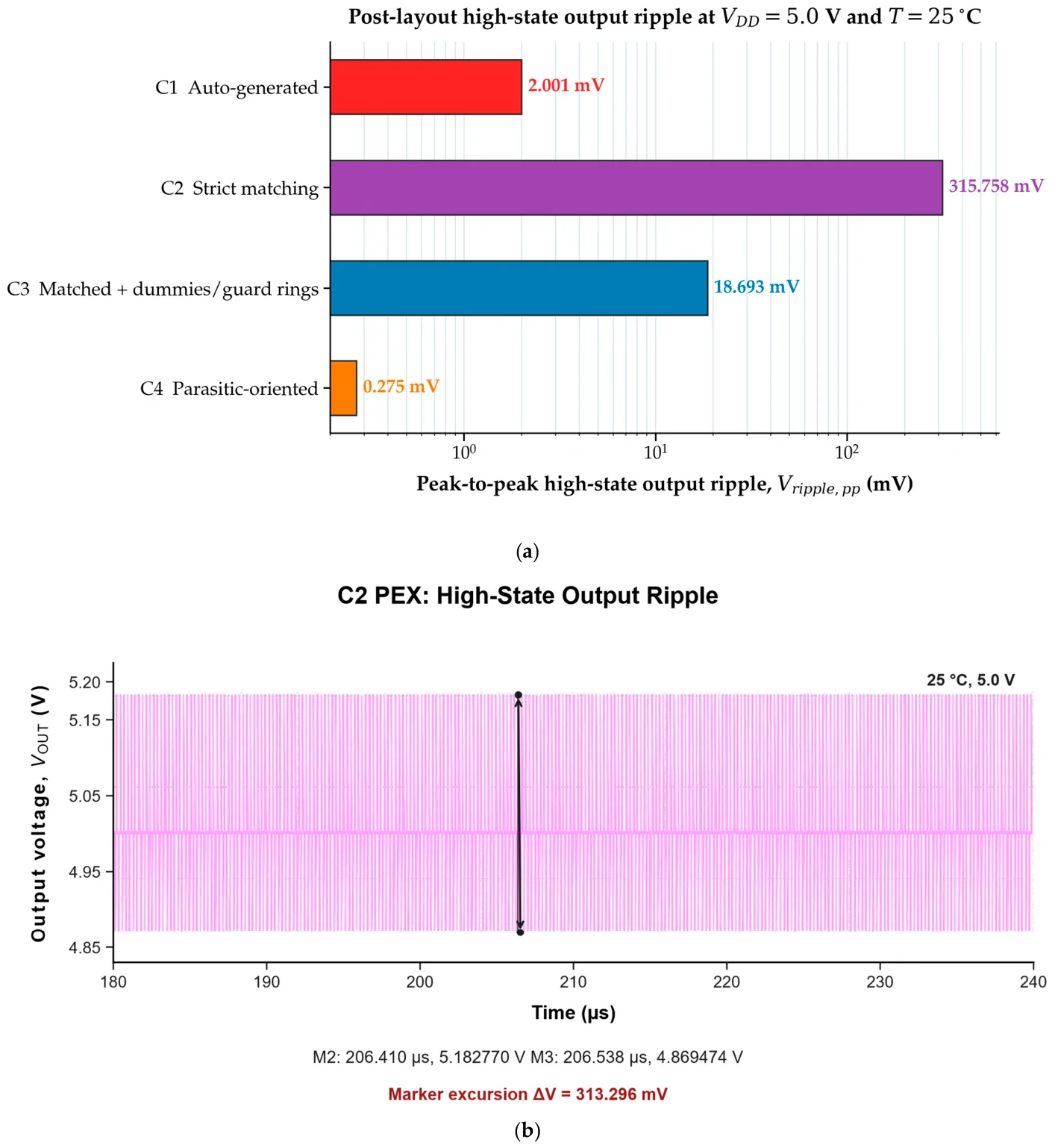
(**a**) Simulated post-layout high-state output ripple for C1–C4 at *V*_DD_ = 5.0 V, *T* = 25 °C, and *C*_L_ = 10 fF. The logarithmic horizontal axis preserves the visibility of C4 while also showing the substantially larger C2 value. (**b**) Measured C2 output waveform in the fixed 180–240 µs high-state measurement interval at 5 V and 25 °C. The Virtuoso markers identify a representative 313.296 mV excursion. The automated peak-to-peak measurement in the 430 µs rerun is 313.647 mV; the figure preserves the original simulated trace.

**Figure 12:**
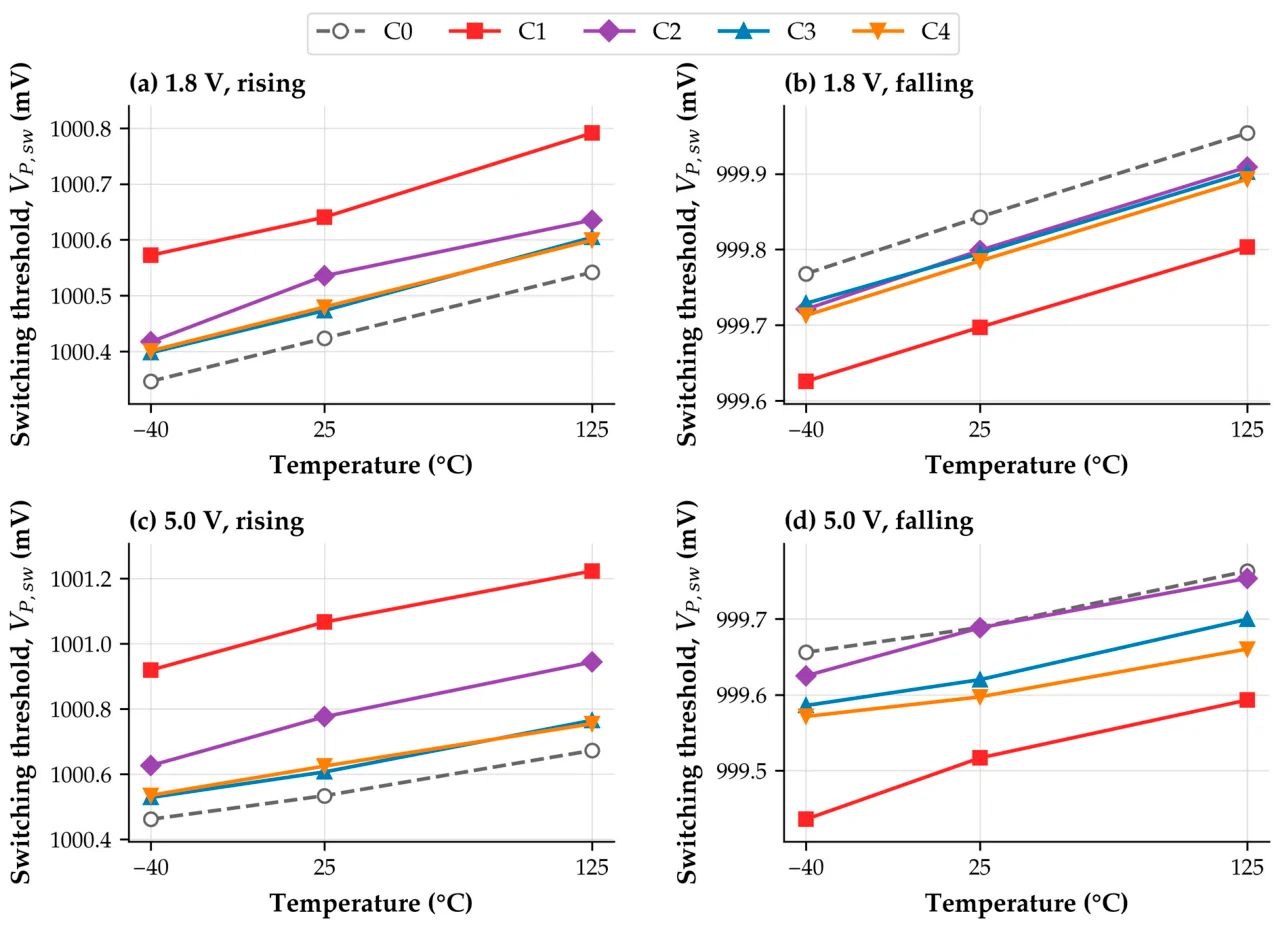
Comparative switching thresholds versus temperature under the common PWL stimulus and 10 fF output load: (**a**) rising threshold at 1.8 V, (**b**) falling threshold at 1.8 V, (**c**) rising threshold at 5.0 V, and (**d**) falling threshold at 5.0 V. Every panel contains C0–C4, with C0 retained as the schematic reference.

**Figure 13:**
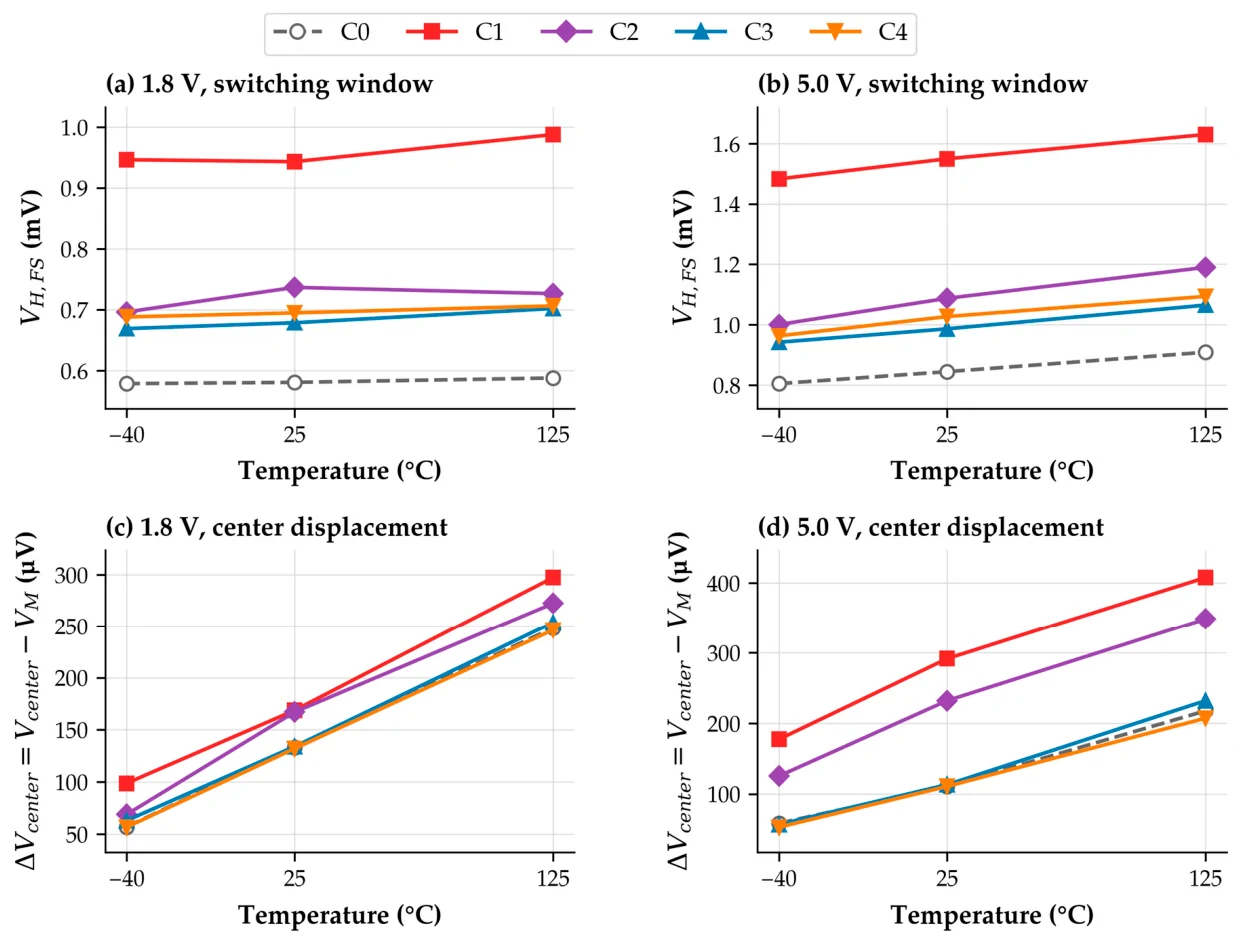
Comparative derived switching metrics versus temperature: (**a**) finite-slew switching window at 1.8 V, (**b**) finite-slew switching window at 5.0 V, (**c**) signed switching-center displacement at 1.8 V, and (**d**) signed switching-center displacement at 5.0 V. Every panel contains the complete C0–C4 nominal-corner dataset.

**Figure 14:**
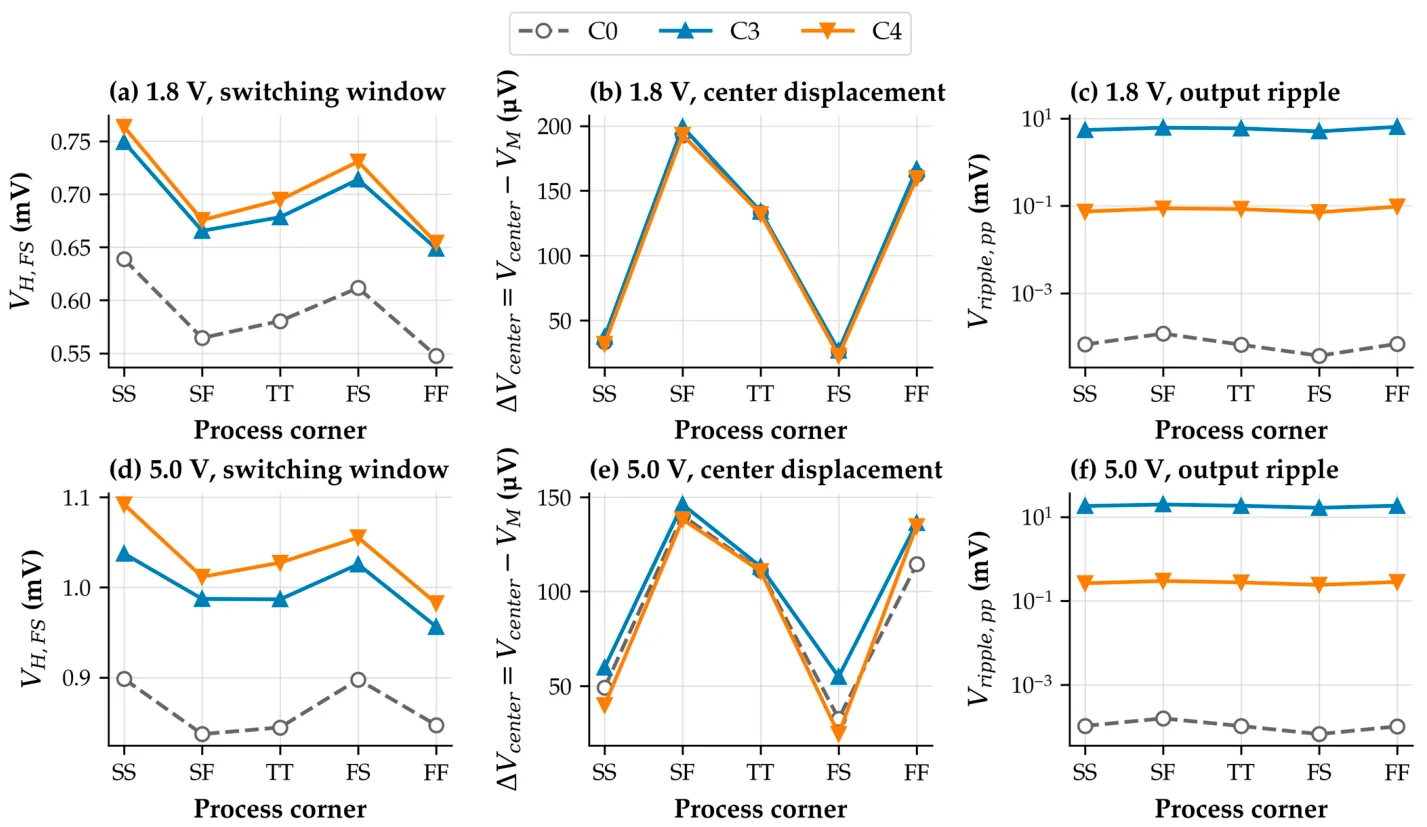
Deterministic process-corner comparison of C0, C3, and C4 at 25 °C: finite-slew switching window, signed switching-center displacement, and settled high-state output ripple at (**a**–**c**) 1.8 V and (**d**–**f**) 5.0 V. The horizontal order is SS, SF, TT, FS, and FF; logarithmic ripple axes retain the visibility of the schematic C0 reference together with both extracted layouts.

**Figure 15:**
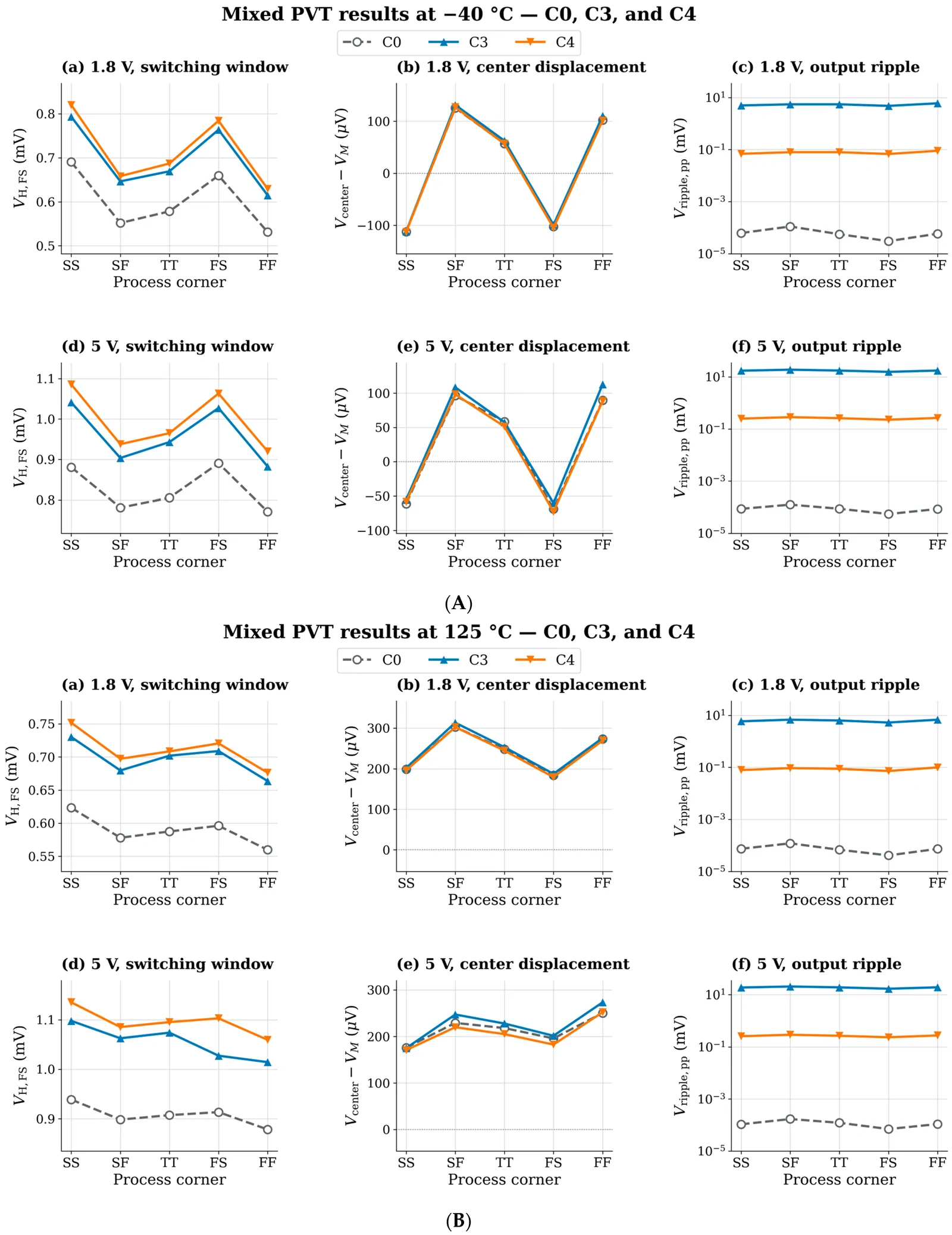
(**A**) Mixed PVT comparison of schematic C0, C3 and C4 at −40 °C for SS, SF, TT, FS, and FF: finite-slew switching window, signed center displacement, and settled high-state output ripple. (**B**) Mixed PVT comparison of schematic C0, C3 and C4 at 125 °C for SS, SF, TT, FS, and FF: finite-slew switching window, signed center displacement, and settled high-state output ripple.

**Figure 16:**
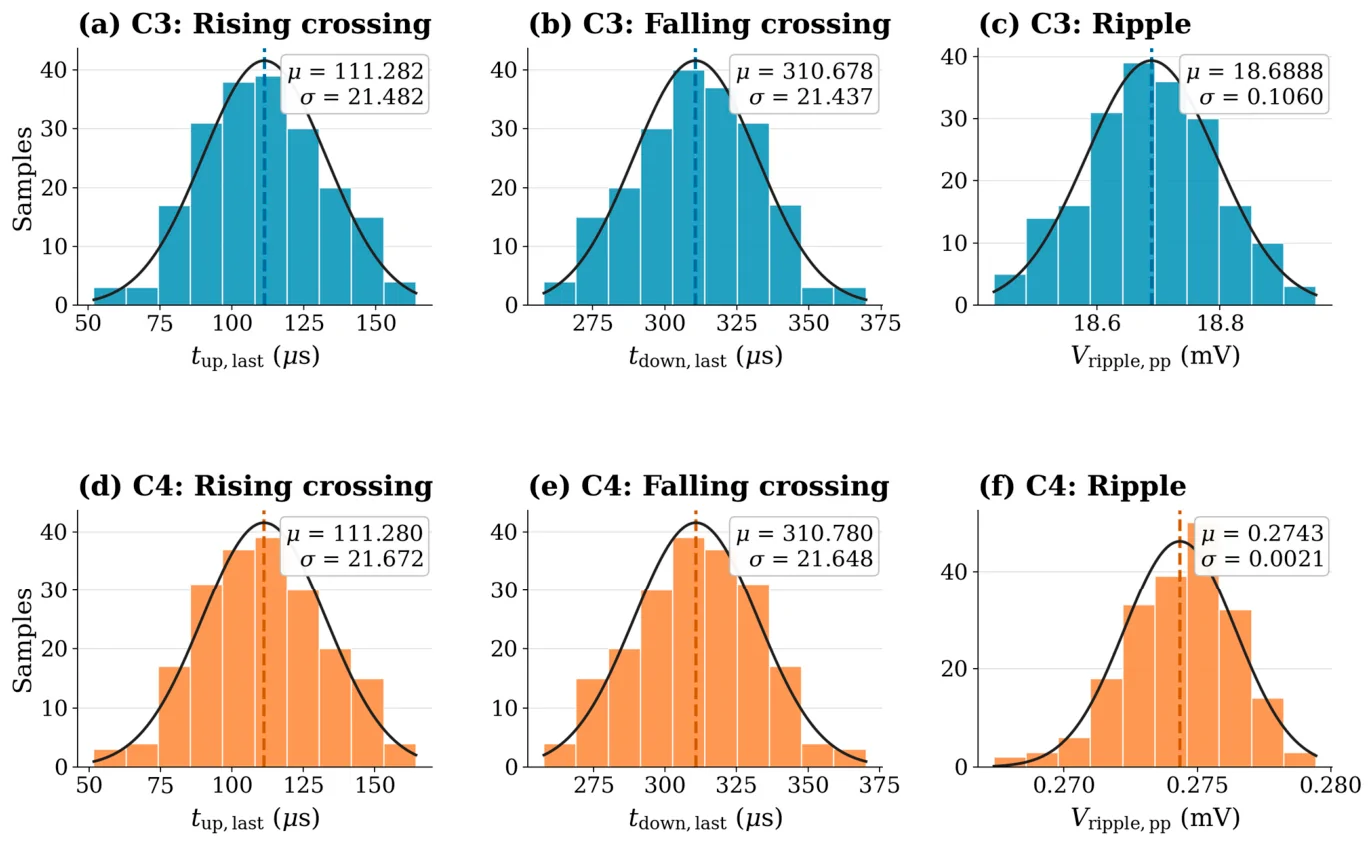
Mismatch Monte Carlo distributions for C3 (**a**–**c**) and C4 (**d**–**f**) at the nominal 5.0 V supply, 25 °C, and the nominal process corner: last rising crossing, last falling crossing, and settled high-state output ripple. Each panel contains 200 samples. Dashed lines show sample means; black curves are normal-reference guides.

**Figure 17:**
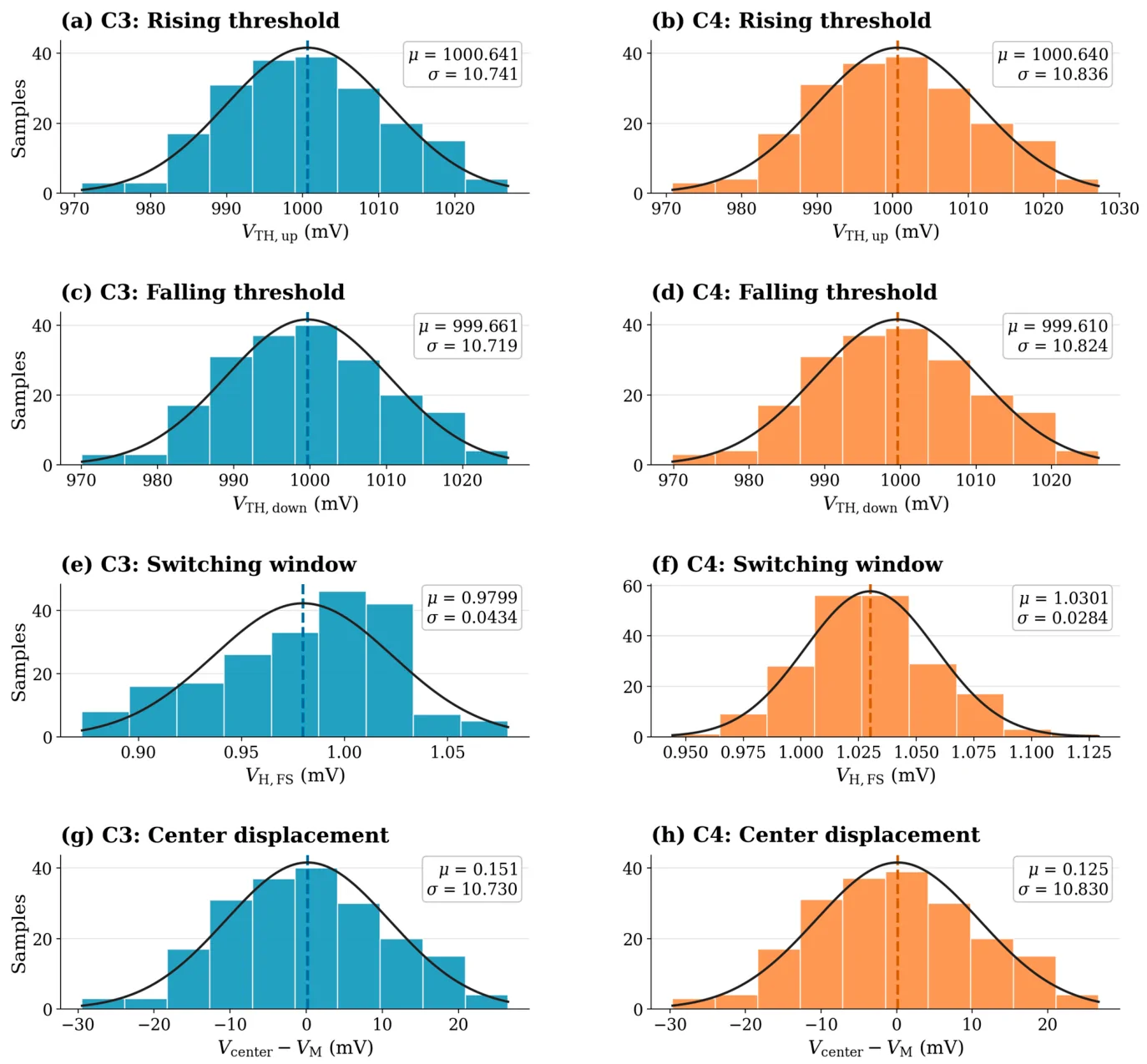
Input-referred mismatch Monte Carlo distributions derived from the 5.0 V sample crossings. C3 occupies the left column (**a**,**c**,**e**,**g**) and C4 the right column (**b**,**d**,**f**,**h**); rows show rising threshold, falling threshold, finite-slew switching window, and signed center displacement. Each panel contains 200 samples. Dashed lines show sample means; black curves are normal-reference guides.

**Table 1:** Schematic transistor W/L and multiplicities.

Devices	Type	W (µm)	L (µm)	m	Circuit Role(s)
M14–M16, M53	PMOS	0.500	0.560	1	Disabled-state pull-ups; final output pull-up
M5, M6, M21	PMOS	1.000	1.000	2	Input active load; second gain stage
M32, M33	PMOS	0.500	2.000	1	Upper regenerative stack
M30, M51	PMOS	0.500	1.000	1	Direct Schmitt pull-up; first-buffer pull-up
M31	PMOS	2.000	0.560	1	Upper feedback-gating device
M13	PMOS	4.000	0.560	1	Enable inverter
M4, M22	NMOS	2.000	2.000	2	Reference branch; second-stage current sink
M12, M54	NMOS	0.500	0.490	1	Bias clamp; final output pull-down
M41	NMOS	1.000	0.490	1	Lower feedback-gating device
M42, M43	NMOS	0.500	4.000	1	Lower regenerative stack
M40, M52	NMOS	0.500	2.000	1	Direct Schmitt pull-down; first-buffer pull-down
M11	NMOS	2.000	0.490	1	Enable inverter
M1, M2	NMOS	1.000	1.000	2	Differential input pair
M3	NMOS	2.000	2.000	5	Differential-pair tail-current sink

**Table 2:** Nominal application and characterization.

Quantity	Nominal Value	Role
Technology class	5 V, thick-oxide bulk CMOS	p-substrate with N-well isolation
Application supply	*V*_DD_ = 5.0 V	Nominal supply of the intended system
Testbench supply	*V*_DD_ = 5.0 V	Main threshold-characterization testbench condition
Application reference	*V*_P_ ≈ 1.04 V	Nominal filtered reference applied to P
Fixed input	*V*_M_ = 1.00 V	Reference used for threshold extraction
Bias current	*I*_BIAS_ = 2 µA	External current-mirror reference
Temperature	T = 25 °C	Nominal characterization temperature

**Table 3:** Controlled physical-layout attributes of variants C1–C4.

Layout Attribute	C1	C2	C3	C4
Placement origin	Virtuoso tool	Manual	Manual	Manual
M1–M2 active-unit pattern	Unconstrained	AB/BA common centroid	AB/BA common centroid	AB/AB; A/B columns
M5–M6 organization	Unconstrained	Matched	Matched	Matched
Schematic W/L/m	Identical	Identical	Identical	Identical
Edge dummy devices	No	No	Yes	Yes
Continuous N+/P+ guard rings	No	No	Yes	Yes
Mandatory well/substrate contacts	Yes	Yes	Yes	Yes
Primary physical objective	Unconstrained baseline	Gradient and symmetry control	Matching plus boundary/isolation control	Shorter critical routes and lower coupling
Controlled inference	Reference baseline	C1→C2: matching	C2→C3: dummies + guard rings, jointly	C3→C4: M1–M2 placement and resulting routing, jointly

**Table 4:** Measured dimensions and physical area of variants C1–C4.

Variant	Measured Outline, W × H (µm)	Bounding-Box Area (µm^2^)	Corner Void (µm^2^)	Occupied Area (µm^2^)	ΔAocc vs. C1
C1	32.42 × 19.06	617.93	0	617.93	Reference
C2	36.015 × 12.55	451.99	23.64	428.35	−30.68%
C3	45.69 × 20.53	938.02	80.80	857.22	+38.73%
C4	45.69 × 20.53	938.02	80.80	857.22	+38.73%

**Table 5:** Compact evaluation plan for C1–C4.

Evaluation	Conditions	Reported Quantities	Purpose
Physical	Same schematic/devices; DRC/LVS; identical R+C+CC extraction	Area; critical-node C; input C/R/coupling balance	Physical cost and parasitic balance
Transient PWL	*V*_P_: 0.95→1.05→0.95 V; *C*_L_ = 10 fF	Last-stable thresholds; *V*_H,FS_; Δ*V*_center_; ripple	Switching and output integrity
Temperature/supply	Same PEX, bias, load, and waveform; −40/25/125 °C; 1.8/5.0 V	Threshold, window, center, and ripple trends	Thermal and supply robustness
Monte Carlo (C3/C4)	200 mismatch samples; nominal process; 5.0 V/25 °C	Timing and voltage-domain mismatch statistics	Mismatch sensitivity

**Table 6:** (**a**) Extracted total capacitance at the critical internal nodes for C1–C4. (**b**) Extracted input-net capacitance, coupling, and distributed route resistance for C1–C4.

(a)				
Internal-Node Capacitance (fF)	C1	C2	C3	C4
DIFF	9.371	6.933	5.427	4.089
DIFFB	4.458	6.858	5.446	4.466
COMPINT	14.703	12.014	12.248	11.190
SCHINT	2.871	5.075	3.795	3.395
OUTB	6.348	4.959	4.379	4.182
OUT	2.878	1.454	1.577	1.496
(**b**)				
**Input-Net Metric**	**C1**	**C2**	**C3**	**C4**
*C*_tot_(P) (fF)	7.5024	4.1865	2.6543	1.5301
*C*_tot_(M) (fF)	2.8941	4.1301	2.9808	1.5326
*|ΔC*_tot_| (fF)	4.6083	0.0564	0.3265	0.0025
CC(P,M) (fF)	0.3227	1.1036	0.5261	0.0962
CC_Σ_(P) (fF)	7.5010	4.1856	2.6537	1.5283
CC_Σ_(M) (fF)	2.8934	4.1294	2.9799	1.5308
ΔCC_in_ (fF)	4.6076	0.0562	0.3262	0.0025
*R*_Σ_(P) (Ω)	18.913	39.364	37.739	25.908
*R*_Σ_(M) (Ω)	18.994	40.103	36.613	25.908
*|ΔR*_in_| (Ω)	0.081	0.739	1.126	<0.001

Note: (**a**) Each value is the corresponding SPEF D_NET total, namely the sum of ground-referenced and incident coupling capacitances defined by Equation (12). OUTB is included because it is both a high-swing internal node and the feedback-control signal of the hysteretic stage. (**b**) CC(P,M) is the direct mutual coupling between the two input nets. The incident sums and their imbalance are defined by Equations (13)–(15); the direct P–M term contributes equally to both sums and cancels in the difference. RΣ is the sum of distributed SPEF resistor elements assigned to an input net and is used as a route-balance descriptor, not as a two-terminal equivalent resistance.

**Table 7:** Nominal finite-slew switching metrics at 25 °C.

Case	*V*_H,FS_ at 1.8 V (mV)	|Δ*V*_center_| at 1.8 V (µV)	*V*_H,FS_ at 5.0 V (mV)	|Δ*V*_center_| at 5.0 V (µV)	Observed Transition
C0	0.580	133.2	0.845	111.0	Clean
C1	0.943	168.7	1.549	291.4	1.8 V: one pre-pulse at 125 °C; 5.0 V: one or two pre-pulses
C2	0.737	167.1	1.087	231.9	Clean
C3	0.678	133.9	0.986	113.1	Clean
C4	0.695	132.1	1.027	110.7	Clean

Note: C0 is the schematic reference, while C1–C4 are the extracted layouts. Every value is calculated from the last stable 50% output crossing at the nominal process corner and 25 °C. The completed table now reports all five cases at both 1.8 V and 5.0 V; the transition classification retains the waveform-level chatter observation for C1 and the clean last-stable behavior observed for the other cases.

**Table 8:** Output-ripple sensitivity when all extracted capacitors connected to the indicated node or group of nodes are zeroed. Each intervention is a separate netlist run. Results for modified netlists are compared to the original 315.758 mVpp result; the rerun values in the final rows check numerical consistency.

Change vs. Baseline	Ripple (mVpp)	C2 Extracted-Netlist Comparison
Reference	315.758	Original C2, original extraction
−53.3%	147.521	Capacitors incident on DIFF set to zero
−25.2%	236.183	Capacitors incident on COMPINT set to zero
−11.8%	278.404	Capacitors incident on OUTB set to zero
−51.6%	152.796	Capacitors incident on SCHINT set to zero
−82.0%	56.878	Capacitors incident on DIFF and DIFFB set to zero
−0.67%	313.647	Original C2 rerun; stop time 430 µs
−0.46%	314.291	Original C2 rerun; stop time 600 µs

## Data Availability

The data presented in this study are available from the corresponding author upon reasonable request. The data are not publicly available because the simulations and extracted layouts rely on proprietary process-design-kit models and technology information subject to confidentiality restrictions.

## Abbreviations

The following abbreviations are used in this manuscript:

CC	Coupling capacitance
CDL	Circuit Description Language
CMOS	Complementary metal-oxide-semiconductor
DC	Direct current
DRC	Design-rule checking
EEPROM	Electrically erasable programmable read-only memory
LDE	Layout-dependent effect
LOD	Length of diffusion
LVS	Layout versus schematic
MOS	Metal-oxide-semiconductor
NMOS	n-channel metal-oxide-semiconductor
NPN	n-p-n bipolar transistor
PDK	Process design kit
PEX	Parasitic extraction
PMOS	p-channel metal-oxide-semiconductor
PNP	p-n-p bipolar transistor
PNPN	p-n-p-n parasitic feedback structure
PWL	Piecewise-linear waveform
R+C+CC	Extraction including resistance, ground-referenced capacitance, and coupling capacitance
SPEF	Standard Parasitic Exchange Format
WPE	Well-proximity effect
